# Establishment of a pharmacokinetics and pharmacodynamics model of Schisandra lignans against hippocampal neurotransmitters in AD rats based on microdi-alysis liquid chromatography-mass spectrometry

**DOI:** 10.3389/fphar.2024.1342121

**Published:** 2024-03-11

**Authors:** Jinpeng Zhang, Xinyuan Cui, Shuo Zhao, Zenghui Chang, Junshuo Zhang, Yufeng Chen, Jiale Liu, Guohao Sun, Yiyuan Wang, Yuanyuan Liu

**Affiliations:** ^1^ Department of Pharmaceutical Analysis, College of Pharmacy, Shandong First Medical University and Shandong Academy of Medical Sciences, Taian, China; ^2^ Qian Xi Nan Maternal and Child Care Hospital, Xingyi, China

**Keywords:** Schisandra chinensis lignans, MD-LC-TQ-MS, Alzheimer’s disease, neurotransmitters, PK-PD

## Abstract

**Objective:** Our previous studies substantiated that the biological activity of Schisandra chinensis lignans during the treatment of Alzheimer’s disease (AD) was mediated by neurotransmitter levels, and 15 of its active components were identified. However, the pharmacokinetic and pharmacodynamic relationship of Schisandra chinensis lignans has been less studied. The objective of this study was to investigate the relationship between the pharmacokinetics and pharmacodynamics of Schisandra chinensis lignans in the treatment of AD, and to establish a pharmacokinetic-pharmacodynamic (PK-PD) model.

**Methods and Results:** Herein, we established a microdialysis-ultra performance liquid chromatography-triple quadruple mass spectrometry (MD-LC-TQ-MS) technique that could simultaneously and continuously collect and quantitatively analyze the active compounds and neurotransmitters related to the therapeutic effects of Schisandra chinensis in awake AD rats. Eight lignans were detected in the hippocampus, and a PK-PD model was established. The fitted curves highlighted a temporal lag between the maximum drug concentration and the peak drug effect. Following treatment, the levels of four neurotransmitters tended to converge with those observed in the sham operation group.

**Conclusion:** By establishing a comprehensive concentration-time-effect relationship for Schisandra chinensis lignans in AD treatment, our study provides novel insights into the *in vivo* effects of these lignans in AD rats.

## 1 Introduction

Schisandra Chinensis is derived from the dried and ripe fruits of the Magnoliaceae plant Schisandra chinensis (Turcz.) Baill. Fructus. It was first recorded in the Shennong Herbal Classic, and it is esteemed for its liver-protective properties. In recent years, pharmacological investigations have unveiled Schisandra lignans’ multifaceted benefits, including enhanced learning and memory capabilities (Olas, 2023), anti-inflammatory actions (Lee et al., 2022), antioxidant effects (Nowak et al., 2019), and more. Schisandra chinensis lignans exhibit neuroprotective attributes by mitigating the expression of phosphorylated tau proteins, abating neuroinflammation arising from overactive microglial cells (Giau et al., 2018; Yang et al., 2019), fostering cellular proliferation, and inhibiting apoptosis (Zhang et al., 2018a). In the preliminary stages, we detected 10 kinds of lignan components of Schisandra chinensis could be detected in the plasma of Alzheimer’s disease (AD) rats (Wei et al., 2019a). On this basis, we carried out further research on the lignan components of Schisandra chinensis and consulted relevant literature (Chen et al., 2018; Chen et al., 2019; Olas, 2023). Through our investigation, we identified a total of 15 components: deoxyschisandrin, schisandrin B, schisandrin C, schisandrol A, schisandrol B, schisantherin A, schisantherin B, gomisin D, angeloylgomisin H, angeloylgomisin Q, gomisin G, gomisin K, schisanhenol, benzoylgomisin H, and gomisin J. Importantly, the utilization of UPLC-Q-TOF-MS for serum and urine metabolomics revealed the potential of Schisandra lignans to regulate neurotransmitter levels in the context of AD treatment (Wei et al., 2019b). Nonetheless, a comprehensive understanding of the dose-response and temporal dynamics of Schisandra lignans in AD therapy remains elusive.

AD is a chronic degenerative disease of the central nervous system that commonly affects the elderly. It is characterized by a decline in memory, as well as mental and emotional abnormalities. Research conducted both domestically and internationally suggests that abnormal deposition of β-amyloid (Aβ) is one of the contributing factors and is a focal point for treatment strategies. The accumulation of Aβ in the brain can lead to the formation of senile plaques and affect the degeneration of neurons, thus inducing the occurrence of AD. (Liu et al., 2015) Specifically, the Aβ (25–35) fragment retains the neurotoxic properties of Aβ and disrupts the balance of excitatory and inhibitory neurotransmission in the brain’s hippocampus. Aβ (25–35) induces excessive Aβ deposition and phosphorylated tau overexpression in a rat model, resulting in neuronal necrosis, upregulation of apoptotic genes, glial cell inflammatory responses, and impaired learning and memory abilities in rats, mimicking AD symptoms. This disruption influences cognitive and memory processes in animals, underscoring the utility of the Aβ (25–35) fragment in establishing AD animal models (Clementi et al., 2005; Mayordomo-Cava et al., 2015).

Microdialysis (MD) involves a minimally invasive sampling approach achieved by implanting a probe equipped with a dialysis membrane at the target position (Wang et al., 2015), which allows substances surrounding the probe to diffuse along the concentration gradient into the probe’s interior. These substances are then transported with the perfusate to a sample collector. Due to the probe’s small size and molecular weight (Jaquins-Gerstl and Michael, 2020), this method causes minimal trauma to the subject, and collected samples can be directly subjected to mass spectrometry analysis without prior treatment (Hou et al., 2022). Another advantageous feature of MD is its ability to enable continuous sampling from the same experimental animal (O'Connell and Krejci, 2022). This capability effectively circumvents the influence of physiological discrepancies among individuals, providing a more accurate representation of *in vivo* drug effects. It is well-established that UPLC-TQ-MS technology amalgamates a high-separation ultra-high-efficiency liquid chromatography system with a highly sensitive triple quadrupole mass analyzer. This combination maximizes efficiency in detecting trace chemical components in blood and tissue.

Consequently, coupling MD with UPLC-TQ-MS technology permits high-throughput screening of active ingredients at targeted sites, further illuminating therapeutic effects and pharmacokinetic traits of drugs within living organisms (Yu et al., 2015). Additionally, this combination enables real-time and precise observations of changes in endogenous active substances (Zhu et al., 2023). The PK-PD binding model integrates the pharmacokinetic properties of a drug within the body with the effects brought by the drug (Li et al., 2022). This model objectively portrays the relationship between concentration, time, and effect. In this regard, Yang Liu et al. (Liu et al., 2019) explored the pharmacokinetics and acetylcholine release effects of ginsenoside Rg1 in the hippocampus of a β-amyloid-induced Alzheimer’s disease rat model using MD and LC-MS/MS techniques. Similarly, Wang Yu et al. (Wang et al., 2020) employed MD-UPLC-TQ-MS technology to quantify active compounds and endogenous neuroactive substances linked to the therapeutic impact of Acanthopanax senticosus in the plasma and hippocampus of rats with ischemic stroke. They established a PK-PD model to delineate the relationship between pharmacokinetics and pharmacodynamics of Acanthopanax senticosus in treating ischemic stroke in rats. Therefore, MD-LC-TQ-MS technology has huge potential for application in PK-PD research.

Herein, we first determined the content of 15 components within Schisandra chinensis lignans. Subsequently, we explored its efficacy in AD treatment through multiple behavioral experiments, HE staining, and immunohistochemistry assays. Finally, leveraging the MD-LC-TQ-MS technique, we established a PK-PD model for Schisandra chinensis lignans in AD rat treatment. We assessed the three-dimensional dynamics of the “time-drug concentration-pharmacodynamic” effect. This investigation elucidates the regulatory impact of Schisandra chinensis lignans on neurotransmitters within the hippocampus of AD rats.

## 2 Materials and methods

### 2.1 Chemicals and materials

*Schisandra chinensis* (Turcz.) Baill. Fructus was procured from Hebei Renxin Pharmaceutical Co., Ltd., China, and authenticated by Prof. Yongxiu Qi (Shandong First Medical University, Taian, China).

Donepezil, deoxyschisandrin, schisandrin B, schisandrin C, schisandrol A, schisandrol B, aspartic acid (Asp), glutamic acid (Glu), and taurine (Tau) were sourced from Shanghai Aladdin Biochemical Technology Co., Ltd. Acetylcholine (Ach) was provided by Nantong Feiyu Biological Technology CO., LTD. Schisantherin B, schisanhenol, and biphenyl dimethyl dicarboxylate were purchased from Shanghai Macklin Biochemical CO., LTD. Angeloylgomisin H and gomisin J were obtained from Shanghai Acmec Biochemical Co., Ltd. Angeloylgomisin Q, gomisin G, gomisin K, gomisin D, and benzoylgomisin H were provided by Beijing Zhongke Quality Inspection Biotechnology Co., Ltd. Aβ (25–35) was purchased from Sigma Odrich Trading Co., Ltd. Methanol, acetonitrile, and formic acid were supplied by Thermo Fisher Scientific (China) Co., Ltd. All other reagents used were of analytical grade.

### 2.2 Sample preparation and extraction

Schisandra chinensis was subjected to a microwave-ultrasonic synergistic extraction. The powder of Schisandra chinensis was mixed with 80% ethanol at a solid-liquid ratio of 1:20 and extracted using ultrasonic power of 200 W and microwave power of 150 W for 30 min. The resulting extract was concentrated to achieve 0.5 g crude drug per 1 mL of liquid.

The concentrated solution underwent purification using AB-8 macroporous resin. Elution was performed with a 7-fold volume of 30% ethanol, followed by hydrolysis adsorption with a 4-fold volume of 90% ethanol. The eluent was concentrated to 2 g crude drug·mL^-1^ and then dried at −60°C to obtain freeze-dried Schisandra lignan purification powder, stored at −80°C for future use.

### 2.3 Animals

Healthy male Sprague-Dawley rats (200 ± 20 g) were procured from Jinan Pengyue Experimental Animal Breeding Co., Ltd. The SPF rats were housed under controlled conditions, with a 12-h light/dark cycle, relative humidity of (50 ± 5) %, and room temperature of (23 ± 2) °C. The animal study was reviewed and approved by Animal Experimentation of Shandong First Medical University and Shandong Academy of Medical Sciences.

Following a 1-week acclimation period, the rats were randomly divided into four groups: blank control group (BLA, *n* = 8), model group (ADM, *n* = 8), positive drug donepezil group (DPZ, *n* = 8), and Schisandra chinensis lignans administration group (SCH, *n* = 8). Aβ (25–35) was dissolved in sterile saline to create a 2 µg per µL solution, which was incubated at 37°C in darkness for 72 h and stored at 4°C (Liu et al., 2018). Rats in the ADM, SCH, and DPZ groups were injected with 5 µL of the incubated Aβ (25–35) protein in the hippocampal CA1 region on both sides. The BLA group was injected with an equal amount of saline. After 2 weeks of modeling, the SCH group was administered 0.55 g Schisandra chinensis per 1 kg of rats, the DPZ group received 0.91 mg donepezil per 1 kg of rats, while the BLA and ADM groups were given an equivalent volume of distilled water. Gavage was performed once daily for 8 weeks.

### 2.4 Measurement of 15 components in the purified lignans of schisandra chinensis

A reference solution of each of the 15 lignan components was prepared and then mixed with different concentration series of the mixed reference solution. All reference solutions were filtered through a microporous filter membrane. The Schisandra lignans purification powder from Section 2.2 was transformed into a solution using methanol and filtered similarly to obtain the test solution. Using the UPLC method, content determination and methodological assessments, including system suitability, linearity, precision, reproducibility, stability, and spiked recovery, were conducted.

UPLC separation of the substances from Schisandra chinensis lignans was carried out on an Ultimate^®^ UHPLC XB-C18 column (1.8 µm, 2.1 × 100 mm) maintained at 35°C with a flow rate of 0.3 mL per min. The mobile phases comprised 0.1% formic acid in water (A) and 0.1% formic acid in acetonitrile (B). The elution gradient of B was as follows: 0–0.2 min, 45%–53% B; 0.2–8 min, 53%–55% B; 8–13 min, 55%–95% B; 13–16 min, 95% B; 16–18 min, 95%–45% B; and 18–20 min, 45% B.

### 2.5 Serum biochemical indexes

The contents of inducible nitric oxide synthase (iNOS), total nitric oxide synthase (TNOS), glutathione peroxidase (GSH-Px), interleukin-6 (IL-6) and interleukin-1β (IL-1β) in rat serum were determined according to the instructions of the kit.

### 2.6 Behavioral test

Following 8 weeks of intragastric administration, a battery of behavioral experiments was conducted to assess the learning and memory capabilities of the rats across different groups. The Y-maze, novel object recognition, active avoidance, and Morris water maze tests were employed.

In the Y-maze experiment (Subaiea et al., 2013), the rats were allowed to move freely within an equilateral triangle area for 10 min. The number of times each rat entered the three arms and the sequence of their entries were recorded. The correct alternating behavior, defined as entering three different arms consecutively, was noted, and the alternation ratio was calculated using the formula: Alternation ratio = (correct alternating behavior/total number of arm entries−2).

For the novel object recognition experiment (Ameen-Ali et al., 2019), rats underwent three stages–adaptation, training, and memory—within an uncovered cubic box. During adaptation, rats moved freely for 5 min without any objects in the box. During the training phase, rats were allowed to explore the box containing two identical objects for 5 min. In the memory phase, one of the objects was replaced with a different one, and the rats’ behavior within 5 min was observed. The results were expressed as the recognition index, calculated as follows: Recognition index = (exploration time of the new object/exploration time of both new and old objects).

The active avoidance experiment (Huang et al., 2021) involved placing rats in a well-lit enclosure with consistent conditions. Across a 3-day period, the rats were given a 90-s window of unrestricted movement on the initial day. Upon entering a dark enclosure on the second day, they encountered electric shocks before being removed. Finally, on the third day, the rats were again allowed free movement, and their escape latency and path from the illuminated enclosure to their first entry into the dark enclosure were documented.

In the Morris water maze experiment (Chiroma et al., 2019) for spatial navigation assessment, rats were placed in the maze from each quadrant to locate a platform within 120 s. If unsuccessful, they were guided to the platform and allowed to stay 10 s before removal. The time taken from start to completion was noted as the escape latency. The subsequent spatial exploration phase involved removing the platform and allowing the rats to explore the maze from the farthest point of the platform. The rats’ trajectories were recorded for 120 s to analyze the time spent in the quadrant containing the platform and the number of platform crossings.

### 2.7 Histological staining and immunohistochemistry assays

Following the behavioral experiments, rats were anesthetized and euthanized. Whole brains were collected and fixed in neutral paraformaldehyde for at least 24 h. Sections (4 μm) were prepared after dewaxing with various alcohol and xylene gradients. These sections were then embedded in paraffin, placed in warm water at 40°C, dried, and deparaffinized for subsequent HE staining and immunohistochemical analysis.

### 2.8 Microdialysis experiments

#### 2.8.1 Preparation of standards

Precisely weighed amounts of deoxyschisandrin, schisandrin B, schisandrin C, schisandrol A, schisandrol B, Schisantherin B, schisanhenol, Angeloylgomisin H, gomisin J, Angeloylgomisin Q, gomisin G, gomisin K, gomisin D, and benzoylgomisin H were dissolved in methanol to create a single standard stock solution of 1 mg mL^−1^, stored at −20°C. This solution was diluted by artificial cerebrospinal fluid (aCSF) into a mixed solution of 1000, 500, 100 ng mL^−1^ for the determination of probe recovery experiment. A series of concentrations were prepared by diluting 1 μg mL^−1^ mixed reference solution with aCSF to determine the standard curve.

Precision-weighed solutions of glutamic acid, aspartic acid, taurine, and acetylcholine chloride were prepared in the same manner as the mixed standards.

#### 2.8.2 Microdialysis system

The microdialysis system consisted of a CMA402 syringe pump, a CMA120 system for freely moving animals, and MAB85 refrigerated Fraction Collector. Collection from the hippocampal region was conducted using CMA12 Elite.

#### 2.8.3 Preparation of perfusion fluid

Artificial cerebrospinal fluid was used as the perfusate. aCSF was formulated using ultrapure water and contained 2.0 mM Na_2_HPO_4_, 10 mM MgCl_2_, 2.7 mM KCl, 145 mM NaCl, and 1.2 mM CaCl_2_, adjusted to pH 7.4 with HCl.

#### 2.8.4 The *in vitro* recovery of the probe

To determine the *in vitro* recovery of the probe, a mixed reference solution containing the 15 lignan components and a mixed standard solution of 4 neurotransmitters at concentrations of 100, 500, and 1000 ng mL^−1^ were employed. Different perfusion rates (1.0, 2.0, and 3.0 μL min^−1^) were tested by immersing the connected probe in the solutions. Each concentration’s standard solution was allowed to equilibrate for 60 min before collecting samples in triplicate. These collected dialysate samples were then analyzed using UPLC-TQ-MS to calculate recovery rates at various flow rates. The probe recovery rate was determined and calculated at the optimal flow rate for different standard solution concentrations.

#### 2.8.5 Preparation of the microdialysate sample

After 8 weeks of intragastric administration, hippocampal dialysates were collected from rats for pharmacokinetic and PK-PD studies. The rats were anesthetized and brain probes were inserted into the rat hippocampus (A: −3.0 mm, L: −2.0 mm, V: −2.6 mm) after cannula implantation, which were secured with dental adhesive. aCSF was perfused at a rate of 2 μL min^−1^ for 90 min, followed by the collection of a 20-min blank dialysate as baseline. Subsequently, all rats were administered Schisandra lignans via intragastric administration, and samples were collected at 20-min intervals over a total of 480 min. These samples were then directly analyzed using UPLC-TQ-MS.

### 2.9 Instruments and LC-MS/MS conditions

Microdialysis samples were analyzed using a 1290Ⅱ ultra-high performance liquid chromatograph (Agilent) and a 6470A triple quadrupole mass spectrometer (Agilent). The liquid phase determination conditions for Schisandra lignans were consistent with those described in Section 2.4. For the neurotransmitter substances, UPLC separation was carried out on a Venusil ASB C18 (5 μm, 4.6 × 250 mm) column maintained at 35 °C and with a flow rate of 0.5 mL min^−1^. Mobile phases consisted of 0.12% formic acid-water (A) and 0.06% formic acid-(80% methanol-water) (B). The elution gradient of B was as follows: 0–2 min, 20%–22% B; 2–8 min, 22%–24% B; 8–9 min, 24%–100% B; 9–14 min, 100% B; 14–15 min, 100%–20% B; 15–18 min, 20% B.

Mass spectrometry analysis utilized an AJS ESI jet point spray ionization source. In the multiple reaction monitoring mode (MRM), 15 lignan components and four neurotransmitters were quantified in the positive mode. The parameters were optimized as follows: sheath temperature: 350°C; sheath flow rate: 11 L min^−1^; dry gas temperature: 300°C; dry gas flow rate: 10 L min^−1^; capillary voltage: 4,000 V; nozzle voltage: 2000 V; nebulizer pressure: 40 Psi; EMV: 200 V. The specific mass spectrometry parameters can be found in Supplementary Table S1.

### 2.10 Method validation

#### 2.10.1 Selectivity

Specificity was assessed by comparing UPLC-TQ-MS chromatograms of all analytes in blank artificial cerebrospinal fluid, mixed standards in blank artificial cerebrospinal fluid, and hippocampal dialysate samples taken 120 min after administration.

#### 2.10.2 Linearity, LOD and LOQ

The mixed standard solution from Section 2.8.1 was analyzed according to the conditions in Section 2.8. A linear regression equation was derived using concentration as the abscissa and peak area as the ordinate. The limit of detection (LOD) was defined as the concentration resulting in a signal-to-noise ratio (S/N) of ≥3, and the limit of quantification (LOQ) was determined with an S/N ratio of ≥10.

#### 2.10.3 Accuracy and precision

The mixed standard solution from Section 2.8.1 was diluted into low (3 times the lower limit of quantitation), medium (40% upper limit of quantitation), and high (80% upper limit of quantitation) quality control solutions. Intra-day and inter-day precision and accuracy were assessed over three consecutive days.

#### 2.10.4 Matrix effect

Low, medium, and high-concentration reference solutions were prepared using aCSF and ultrapure water as solvents. Peak areas were recorded after UPLC-TQ-MS determination, and the matrix effect was evaluated using the ratio of analyte peak area in blank brain dialysate to peak area in aCSF.

### 2.11 Statistical analysis

All data were analyzed using SPSS 26.0 statistical software. For variance analysis, the Least Significant Difference (LSD) and Student-Newman-Keuls (SNK) methods were employed, with a test level of α = 0.05. Non-compartmental model analysis (NCA) in WinNonLin 8.1.0 was used to calculate PK and PD parameters for Schisandra chinensis, and results were used to establish the PK-PD model.

## 3 Results

### 3.1 Methodological investigation and content determination of 15 chemical substances in the purified lignans of Schisandra chinensis

**FIGURE 1:**
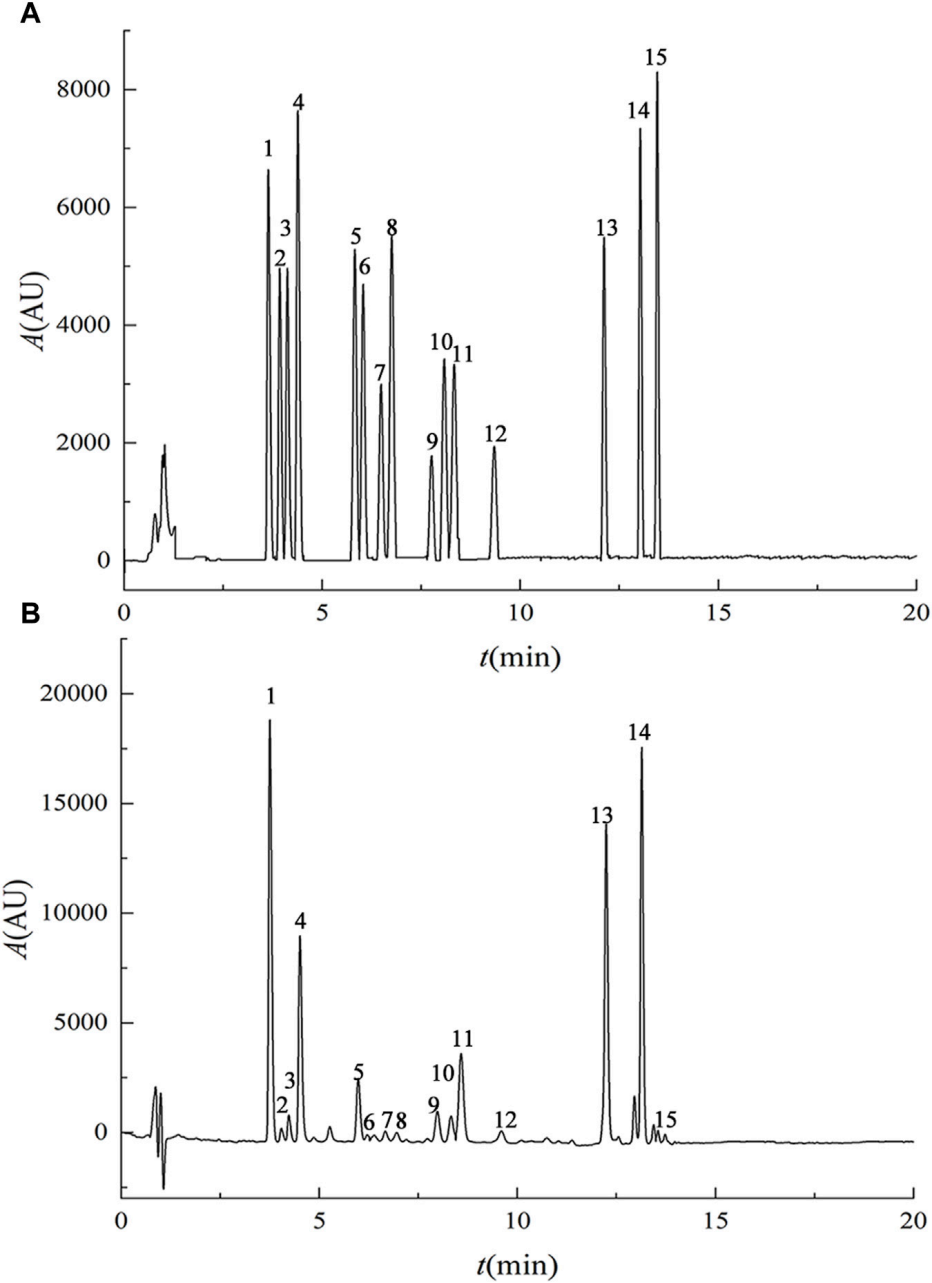
UPLC chromatograms of mixed reference substances **(A)** and purified lignans from Schisandra chinensis **(B)**. Note: 1. schisandrol A, 2. gomisin D, 3. gomisin J, 4. schisandrol B, 5. angeloylgomisin H, 6. benzoylgomisin H, 7. angeloylgomisin Q, 8. gomisin G, 9. gomisin K 10. schisantherin A 11. schisantherin B 12. schisanhenol 13. deoxyschisandrin 14. schisandrin B 15. schisandrin C.

Results from the methodological investigation (Figure 1) revealed distinct chromatographic peaks for the 15 lignan components, displaying symmetrical peak shapes and clear separation. The method exhibited excellent linearity, reproducibility, stability, and recovery characteristics, meeting instrumental precision requirements. Content determination of the purified Schisandra lignans revealed the following percentages for various components: deoxyschisandrin (4.40%), schisandrin B (5.11%), schisandrin C (0.18%), schisandrol A (12.35%), schisandrol B (3.02%), schisantherin A (0.83%), schisantherin B (2.84%), gomisin D (0.57%), angeloylgomisin H (2.43%), angeloylgomisin Q (0.76%), gomisin G (0.43%), gomisin K (2.66%), schisanhenol (1.06%), benzoylgomisin H (0.29%), and gomisin J (0.75%).

### 3.2 Method validation

#### 3.2.1 Selectivity

**FIGURE 2:**
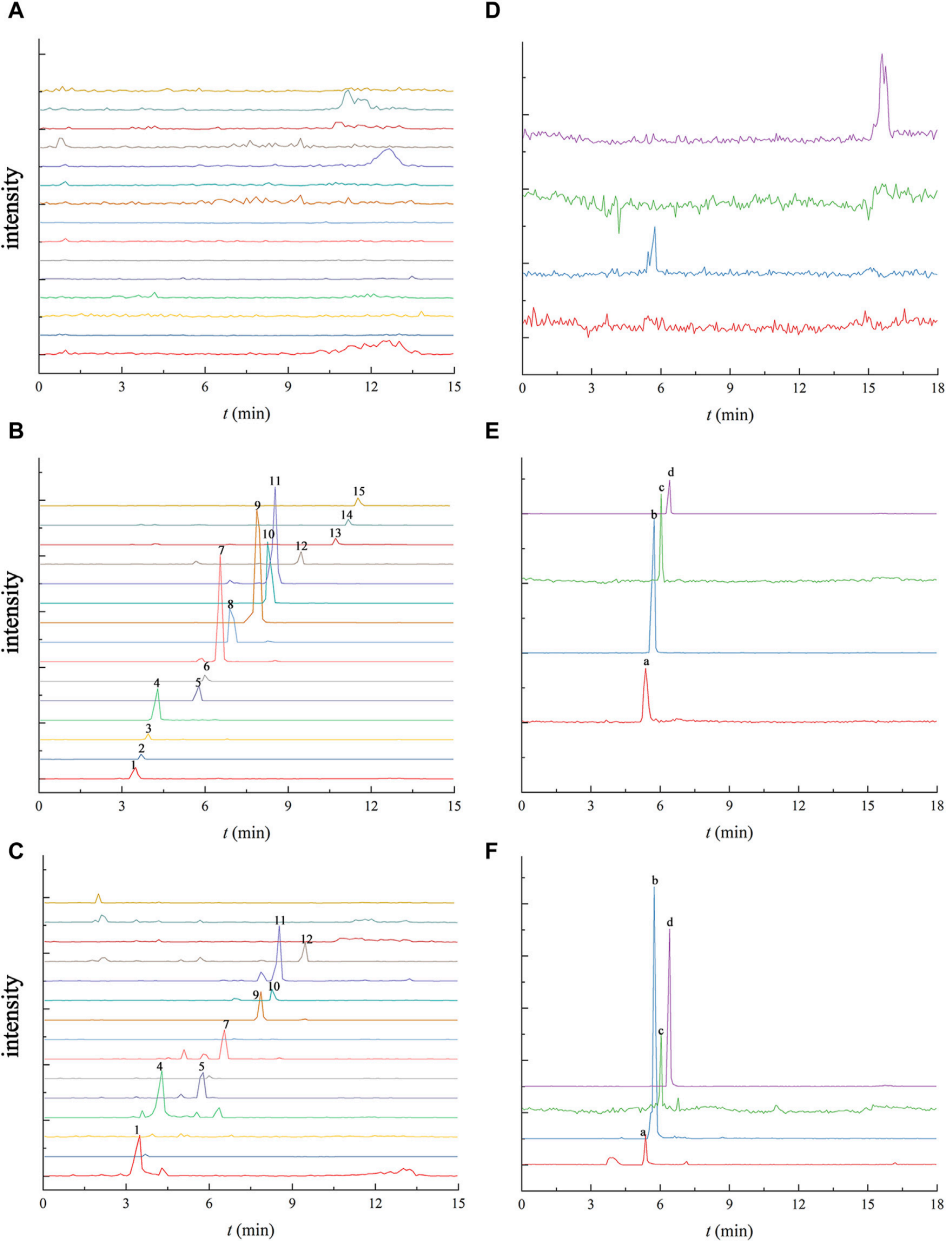
UPLC-TQ-MS chromatogram of 15 Schisandra lignans and 4 neurotransmitters in the blank aCSF **(A and D)**, the blank aCSF spiked with the mixed standard solution **(B and E)** the and the collected microdialysis sample of the hippocampus at 120 min after oral administration of Schisandra lignans **(C and F)**. Note: 1. schisandrol A, 2. gomisin D, 3. gomisin J, 4. schisandrol B, 5. angeloylgomisin H, 6. benzoylgomisin H, 7. angeloylgomisin Q, 8. gomisin G, 9. gomisin K, 10. schisantherin A, 11. schisantherin B, 12. Schisanhenol, 13. deoxyschisandrin, 14. schisandrin B, 15. schisandrin C, a. Ach, b. Glu, c. Asp, d. Tau.

Figure 2 illustrates the absence of significant interference, comparing the chromatograms of the mixed standard solution in aCSF with the hippocampus dialysate chromatogram after 120 min of administration, which indicated that the dialysate substances did not interfere with one another, affirming the feasibility of the determination method. In Figure 2C, gomisin D, gomisin J, benzoylgomisin H, gomisin G, deoxyschisandrin, schisandrin B and schisandrin C were not detected, that may be caused by the low content of these ingredients in Schisandra chinensis and obstruction of the blood-brain barrier. Deoxyschisandrin, schisandrin B and schisandrin C are more lipophilic compared to the other ingredients making them difficult to detect in aCSF with water as the main solvent.

#### 3.2.2 Linearity, LOD and LOQ

Linear regression equations were established using the concentration of the 15 lignans and 4 neurotransmitters as the abscissa, with peak area as the ordinate. Table 1 presents the results, indicating favorable linearity within the standard curve range, with correlation coefficients exceeding 0.9924.

**TABLE 1:** Linear ranges, regression equations, correlation coefficients *R* ^2^, LOD and LOQ of 15 lignans and 4 neurotransmitters.

Component	Linear ranges (ng/mL)	Regression equations	*R* ^2^	LOD (ng/mL)	LOQ (ng/mL)
schisandrol A	1–100	y = 63.058x-66.836	0.9986	0.2	0.7
	10–1000	y = 63.012x+137.11	0.9946		
gomisin D	0.1–50	y = 57.309x-19.468	0.9985	0.18	1
	10–1000	y = 59.265x-836.26	0.9924		
gomisin J	0.1–50	y = 57.03x-13.132	0.9989	0.08	0.1
	10–1000	y = 61.148x-525.48	0.9988		
schisandrol B	0.1–50	y = 429.93x+58.075	0.9994	0.016	0.1
	10–1000	y = 441.42x-4732.1	0.9942		
angeloylgomisin H	1–75	y = 37.544x+59.028	0.9949	0.08	0.2
	10–1000	y = 37.175x+125.24	0.9974		
benzoylgomisin H	1–75	y = 22.509x+56.472	0.9969	0.25	1
	10–1000	y = 25.906x-249.93	0.9944		
angeloylgomisin Q	0.1–50	y = 137.37x+125.96	0.9971	0.04	0.1
	10–1000	y = 129.92x+1492.8	0.9968		
gomisin G	0.1–50	y = 80.523x+19.909	0.9992	0.05	0.1
	10–1000	y = 84.298x-484	0.9981		
gomisin K	0.01–25	y = 2865.3x-84.865	1	0.001	0.01
	10–1000	y = 3103x-896.21	0.9982		
schisantherin A	0.1–50	y = 422.1x-25.487	0.9999	0.01	0.1
	10–1000	y = 406.2x+3724	0.9992		
schisantherin B	0.1–50	y = 106.77x+60.297	0.9981	0.016	0.09
	10–1000	y = 108.48x-10.777	0.9995		
schisanhenol	0.1–50	y = 329.44x-108.29	0.9984	0.015	0.075
	10–1000	y = 321.62x+527.08	0.999		
deoxyschisandrin	0.1–50	y = 271.16x-64.642	0.9926	0.001	0.1
	10–1000	y = 252.16x+1169.8	0.9994		
schisandrin B	0.1–50	y = 406.79x-167.02	0.9996	0.008	0.08
	10–1000	y = 415.39x-4326.6	0.9985		
schisandrin C	1–75	y = 12.851x+26.755	0.9976	0.05	0.5
	10–1000	y = 12.678x+96.846	0.9989		
Asp	10–300	y = 79.457x+3061.5	0.9945	0.18	7.69
Glu	20–400	y = 299.72x+5815.8	0.9971	0.5	2
Tau	20–400	y = 123.18x+1036.6	0.9987	0.04	1
Ach	0.05–20	y = 6949.8x-919.97	0.9953	0.01	0.04
	10–300	y = 6560.9x+3650.7	0.9999		

#### 3.2.3 Accuracy and precision

Supplementary Table S2 outlines the precision and accuracy outcomes for the 15 lignan components and 4 neurotransmitters at low, medium, and high concentrations. Intra-day precision (RSD) ranged from 0.81% to 12.38%, and accuracy (RE) ranged from −6.63% to 9.90%. Inter-day RSD ranged from 0.31% to 7.18%, and RE ranged from −6.31% to 9.62%. These results met the requirements for biological sample determination, indicating good instrumental precision.

#### 3.2.4 Matrix effect

Matrix effect results are displayed in Supplementary Table S2, showing a range of 92.24% ± 4.04% to 106.85% ± 7.49%, which suggests the minimal impact of the matrix on the test substances.

### 3.3 *In vitro* recovery of the microdialysis probes

**FIGURE 3:**
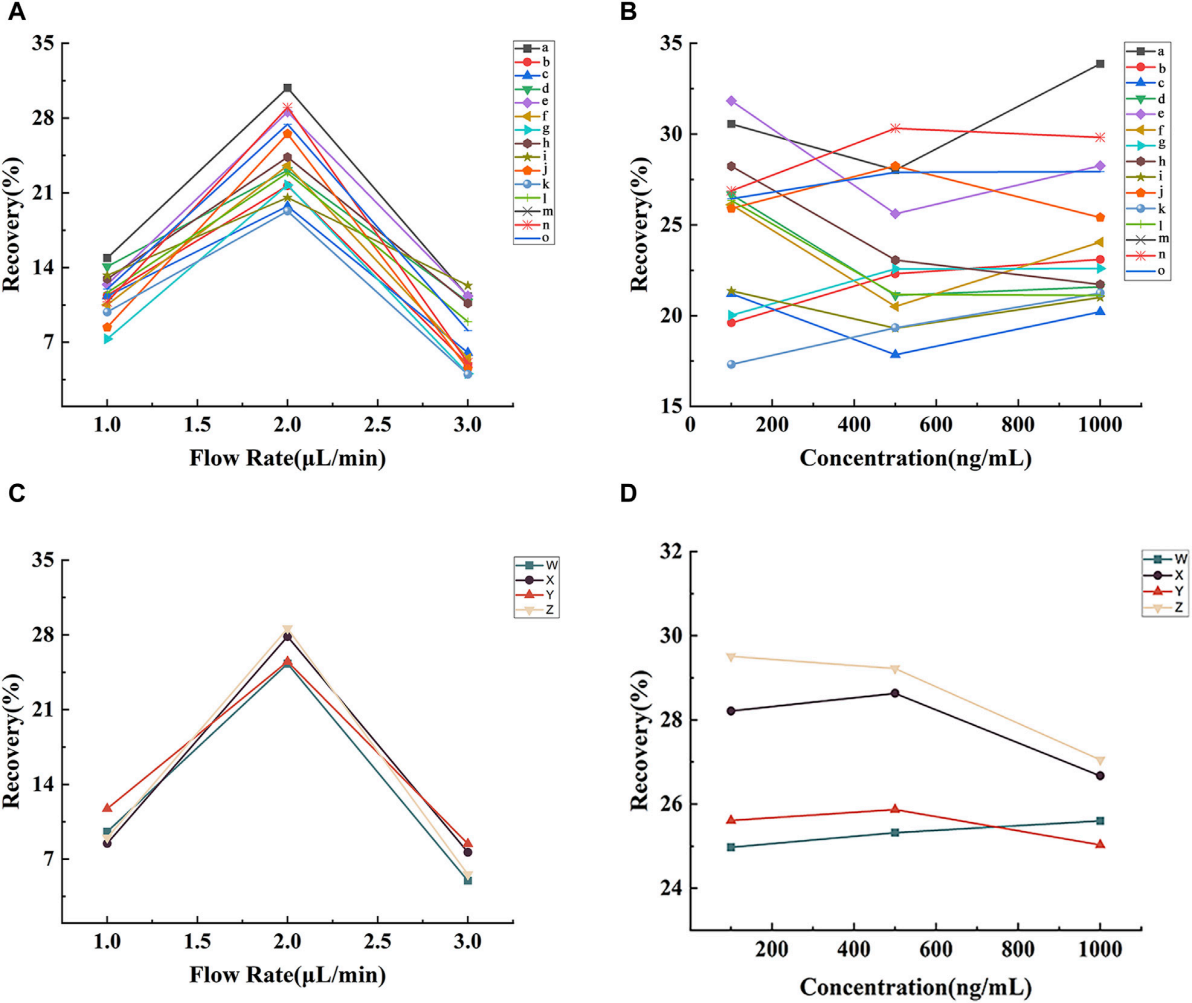
Effects of perfusion rate and concentration of lignans **(A and B)** and neurotransmitters **(C and D)** on the recovery of microdialysis probes *in vitro*. Note: (a) benzoylgomisin H, (b) schisandrin C, (c) schisandrol A, (d) schisandrol B, (e) angeloylgomisin H, (f) angeloylgomisin Q, (g) schisanhenol, (h) gomisin D, (i) gomisin G, (j) gomisin J, (k) gomisin K, (l) deoxyschisandrin, (m) schisandrin B, (n) schisantherin A, (o) schisantherin B, (W) Asp, (X) Glu, (Y) Tau, (Z) Ach.

The outcome of the recovery rate analysis for the lignan probe is presented in Figures 3A,B. Figure 3A illustrates the considerable impact of perfusion speed on the probe’s recovery rate. Notably, at a perfusion speed of 2.0 μL min^−1^, the probe demonstrated its highest recovery rate, ranging from 19.29% to 30.82%. At this speed, probe recovery rates were assessed at lignan concentrations of 100, 500, and 1000 ng min^−1^, as showcased in Figure 3B, revealing the minimal impact of lignan concentration on the probe’s recovery rate.

The recovery results of the neurotransmitter probes are depicted in Figures 3C,D. As depicted in Figure 3C, the probe recovery rates for Asp, Glu, Tau, and Ach were the highest at 2.0 μL min^−1^ (25.30%, 27.84%, 25.50%, and 28.59%, respectively). As shown in Figure 3D, neurotransmitter concentration had limited influence on probe recovery at 2.0 μL min^−1^. Consequently, subsequent microdialysis experiments were conducted at a 2.0 μL min^−1^ perfusion rate.

### 3.4 Pharmacodynamics evaluation

#### 3.4.1 Effects of SCH on serum biochemical indices in AD rats

Studies have shown that the Aβ protein can stimulate the formation of amyloid plaques, activate glial cells to release inflammatory factors (IL-6, IL-1β), lead to neuroinflammation, and accelerate the death of neuronal cells (Guo et al., 2001; Liu et al., 2023). Additionally, the toxic effect of Aβ protein can upregulate the expression levels of total nitric oxide synthase (TNOS) and inducible nitric oxide sythase (iNOS) in rats, resulting in an increase in the production of nitric oxide (NO)(Dubey et al., 2020), affecting the normal stress response and accelerating the death of nerve cells (Bujalska-Zadrożny et al., 2015). At the same time, the excessive deposition of Aβ protein in the brain will also reduce the level of antioxidant enzymes (GSH-Px), disrupting the balance of normal oxidative stress response in the body, thereby inducing free radical damage and aggravating the development of AD (Mao and Reddy, 2011).

**FIGURE 4:**
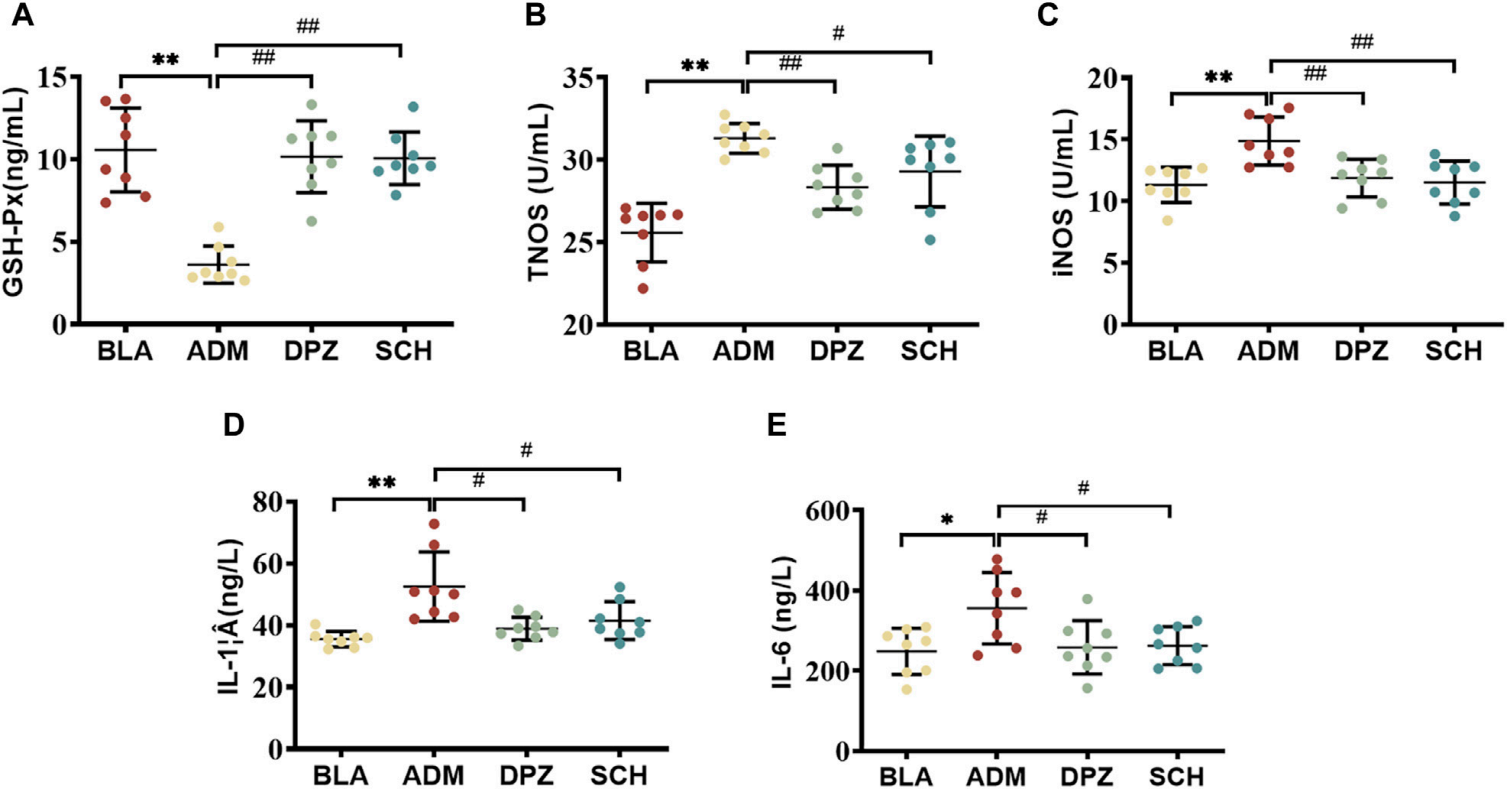
Effects of Schisandra chinensis lignans on **(A)** GSH-Px content, **(B)** TNOS activity, **(C)** iNOS activity, **(D)** IL-1β content, **(E)** IL-6 content in the serum of AD rats. Data were presented as the mean ± SD (*n* = 8). **p < 0.01 vs. BLA group; #*p* < 0.05, ##*p* < 0.01 vs. ADM group.

Figure 4 shows that the levels of IL-1β, IL-6 (*p* < 0.05), TNOS and iNOS were significantly increased (*p* < 0.01) in the ADM group compared with the BLA group, indicating that the levels of pro-inflammatory factors in the ADM group were increased, which aggravated the development of AD. After treatment, the levels of these indicators significantly decreased in the DPZ and SCH groups (*p* < 0.01). The results showed that Schisandra lignans combined with donepezil in the treatment of AD rats could reduce the activity of TNOS and iNOS, inhibit the overexpression of IL-1β and IL-6, reduce the body’s redox and inflammatory response, and improve learning and memory dysfunction. In addition, compared with the BLA group, the GSH-Px content in the serum of the ADM group were significantly decreased (*p* < 0.01), compared with ADM group, GSH-Px content in DPZ group and SCH group were significantly increased (*p* < 0.05). The results suggest that the level of the GSH-Px enzyme in the ADM group is lower, and the progression of AD is accelerated. It was found that Schisandra lignans and donepezil can improve the activity of antioxidant enzymes, thereby improving the learning and memory dysfunction of AD rats.

#### 3.4.2 Effects of SCH on the performance of AD rat in multiple behavioral tests

**FIGURE 5:**
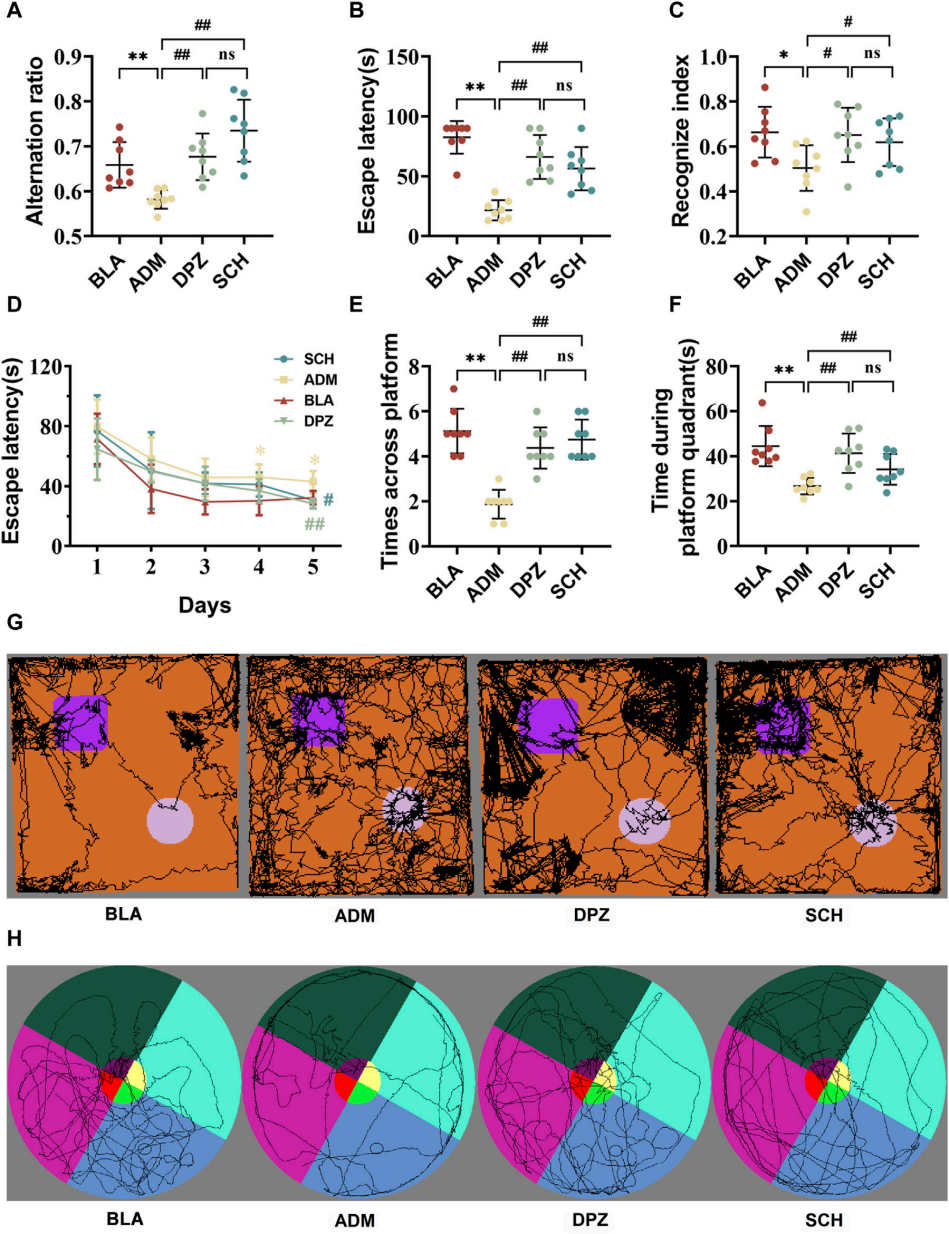
The results of treatment with Schisandra chinensis lignans on multiple behavioral tests. **(A)** Alternating ratio in Y-maze test. **(B)** Escape latency in active avoidance test. **(C)** Recognize index in novel object recognition test. **(D)** Recognition trace of each group of rats in the new object recognition test. **(E)** Number of times the rat crossed platform position and **(F)** time spent in the target quadrant during the Morris water maze test. **(G)** Recognition trace of each group of rats in the new object recognition test. **(H)** Escape latency time of rats in each group. Data were presented as the mean ± SD (*n* = 8). **p < 0.01 vs. BLA group; ##*p* < 0.01 vs. ADM group; ns *p* > 0.05 vs. DPZ group.

Figure 5A demonstrates that the ADM group displayed a significantly reduced alternation ratio compared to the BLA group (*p* < 0.01), indicating impaired short-term memory capacity in AD rats. However, after treatment, the DPZ and SCH groups exhibited significant improvements (*p* < 0.01).

As shown in Figure 5B, the escape latency of the ADM group was notably reduced compared to the BLA group (*p* < 0.01), signifying impaired memory ability concerning punitive events in AD rats. Subsequent treatment in the DPZ and SCH groups led to increased escape latency (*p* < 0.01), indicative of enhanced memory related to punitive events.

Figures 5C,G demonstrate that the new object recognition index of the ADM group was significantly diminished (*p* < 0.05), and the exploration of old and new objects was comparable, suggesting impaired memory capacity in AD rats compared with the BLA group. After treatment, both the DPZ and SCH groups exhibited significantly increased new object recognition indexes (*p* < 0.05), with exploration of new objects surpassing that of old objects.

In the context of the 5-day spatial navigation test (Figures 5D–F,H), the ADM group exhibited the longest escape latency, displaying significant differences from the SCH group on day 4. The SCH group exhibited the steepest decline, leveling off around day 3. The DPZ group closely mirrored the trend of the SCH group, exhibiting a significant difference with the ADM group on day 5. During the spatial exploration stage, compared to the SCH group, the ADM group displayed a substantial decrease in the number of platform crossings and platform quadrant retention time. Their movement trajectories were more evenly distributed across the four quadrants, indicative of impaired spatial learning and memory in AD rats. After treatment, the DPZ and SCH groups demonstrated increased numbers of platform crossings and platform quadrant retention times alongside increased trajectories within the platform quadrant. The therapeutic effects of Schisandra lignans and donepezil showed no significant difference (*p* > 0.05), indicating that they did not show good regularity in the treatment of AD, but they both exerting a positive effect on it.

Collectively, these findings underline the impairment of memory and learning abilities in AD rats. However, both donepezil and Schisandra lignans demonstrated the capacity to enhance the learning and memory abilities of AD rats, thereby exerting a positive effect on AD.

#### 3.4.3 The effect of SCH on hippocampal neuronal morphology

**FIGURE 6:**
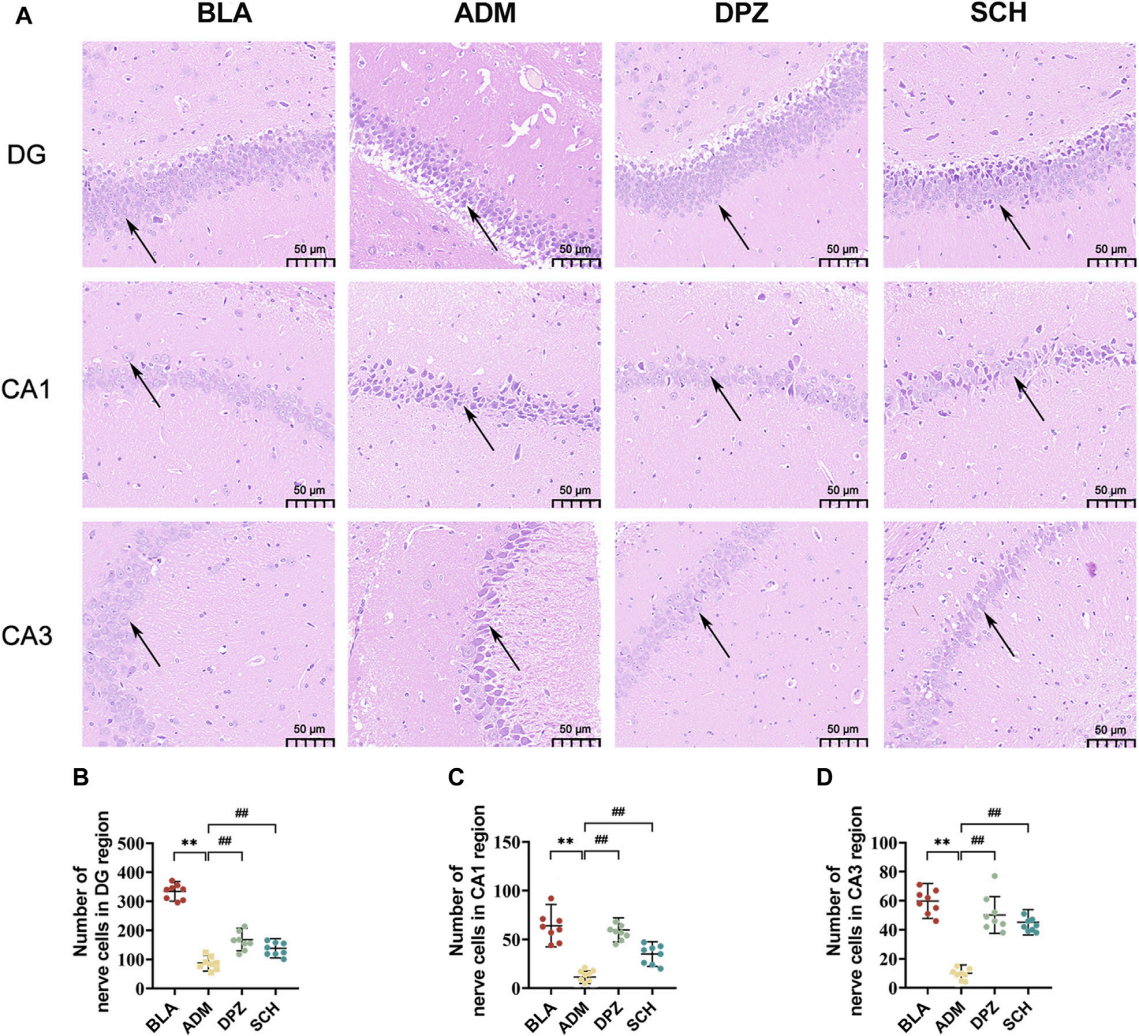
**(A)** HE staining images and **(B–D)** number of nerve cells in DG, CA1 and CA3 subregions of hippocampus of rats in different groups. Scale bars = 50 µm. Results are representative of three times and expressed as the mean ± SD. *p < 0.05, **p < 0.01 vs. BLA group; #*p* < 0.05, ##*p* < 0.01 vs. ADM group.

Figure 6A highlights those rats in the BLA group exhibited lighter staining within the hippocampus, with round-shaped cells, moderate intercellular spacing, distinct and dense cellular distribution, and orderly arrangement, resulting in a structurally complete configuration. In contrast, the ADM group displayed evident AD-related pathological morphology, featuring darker staining, irregular cell forms, enlarged intercellular spaces, a notable occurrence of neuronal necrosis and the number of nerve cells was significantly reduced (*p* < 0.01). Post-treatment with Schisandra lignans showed improvements in the pathological status of AD rats, with reduced atrophic morphological cell counts, approaching normal cell morphology and structure. Additionally, the number of nerve cells (Figures 6B–D) showed a significant increase (*p* < 0.01).

**FIGURE 7:**
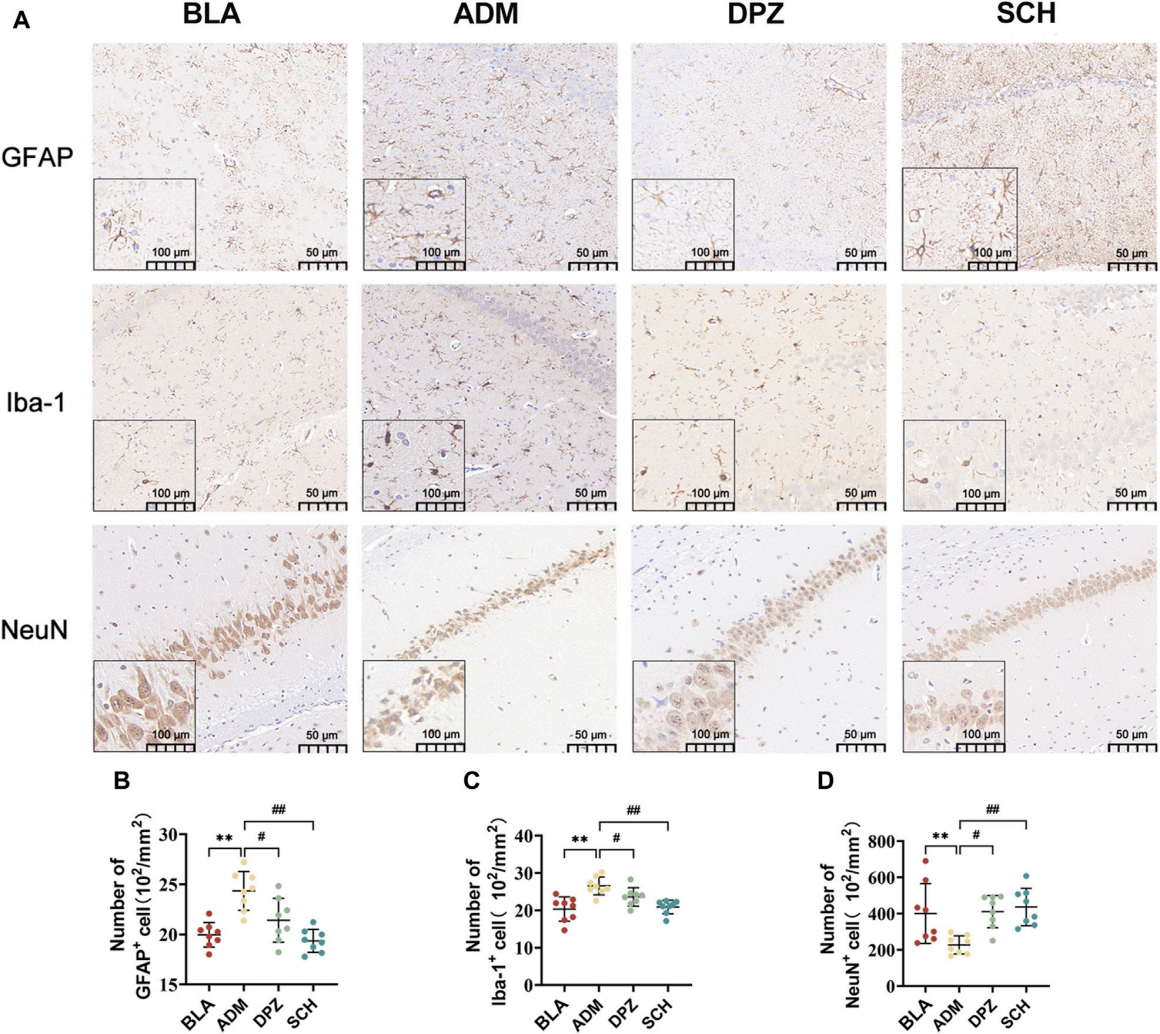
**(A)** The expression images of GFAP, Iba-1 and NeuN in the immunohistochemical experiments in the hippocampus of different groups of rats and **(B–D)** the number of positive cells. Scale bars = 50 µm. Results are representative of three times and expressed as the mean ± SD. *p < 0.05, **p < 0.01 vs. BLA group; #*p* < 0.05, ##*p* < 0.01 vs. ADM group.

As shown in Figure 7A, the ADM group exhibited higher staining intensity, increased positive expression of GFAP and Iba-1, and a substantial increase in positively-stained area (*p* < 0.01) compared with the BLA group, which suggests significant activation of astrocytes and microglia in AD rats, promoting reactive glial cell proliferation, heightened release of inflammatory factors, and consequent neuronal damage. After treatment, the DPZ and SCH groups exhibited less intense staining, with GFAP and Iba-1 positive expression levels approaching those of the BLA group, indicative of alleviated AD conditions.

Furthermore, the ADM group exhibited a lower abundance of NeuN-positive cells and significantly reduced positive area, indicating heightened neural tissue damage and compromised learning and memory abilities in AD rats. Conversely, the BLA and DPZ groups exhibited increased NeuN-positive cell counts (Figures 7B–D) and augmented positive areas, contributing to improved learning and memory impairments in AD rats.

#### 3.4.4 Pharmacodynamic study of neurotransmitters

**FIGURE 8:**
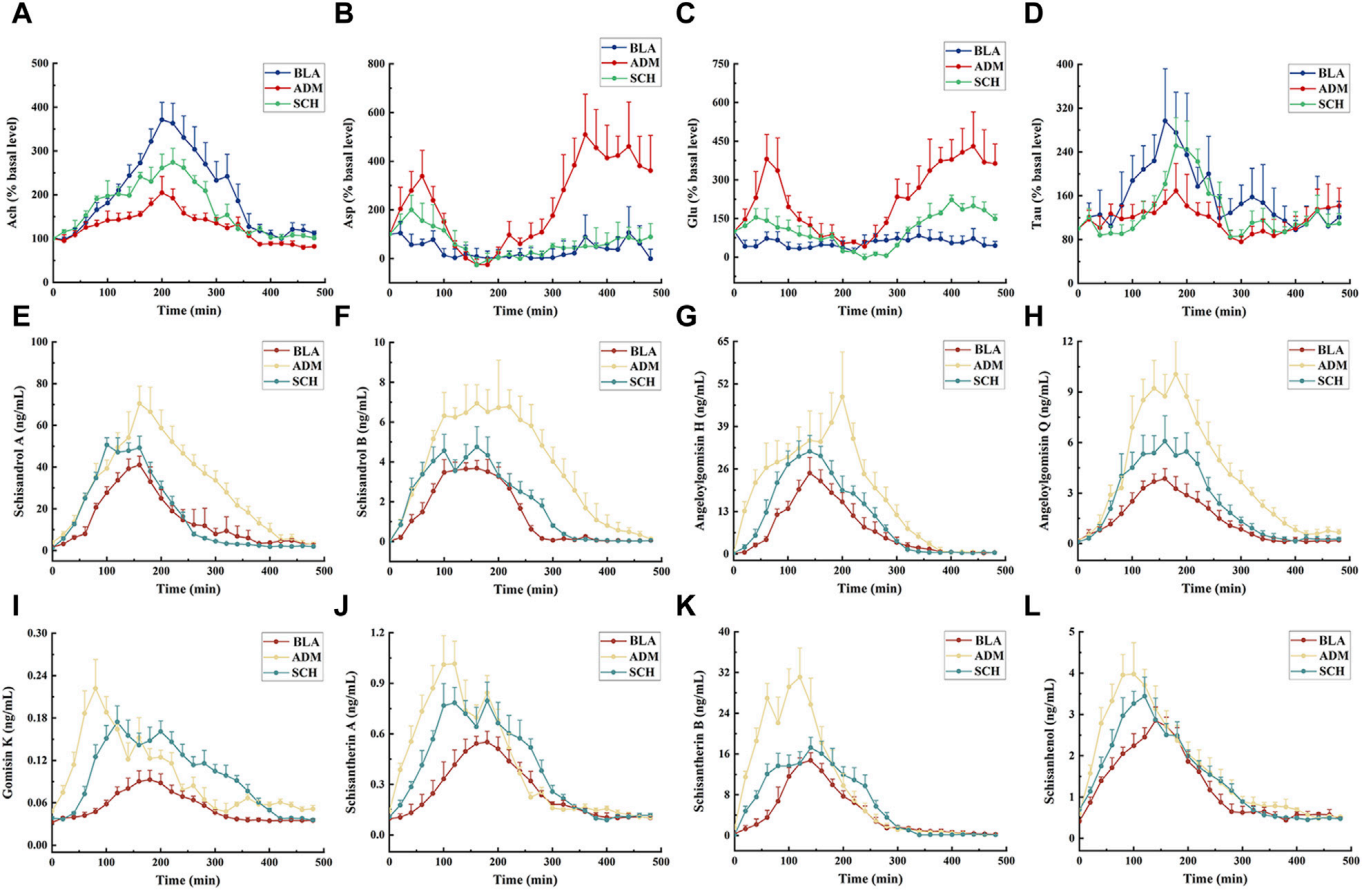
The mean concentration–time curve of 4 neurotransmitters **(A–D)** and 8 lignans **(E–L)** in the microdialysate of hippocampus of rats in different groups after oral administration with purified Schisandra lignans. The drug injection time is 0 min. Asp, Glu, Ach and Tau release expressed as percent change over the first basal sample before administrating and normalized to 100%.

The dynamic changes in four neurotransmitters within the hippocampal microdialysate of rat groups (SCH, ADM, and BLA) were examined using the MD-LC-TQ-MS technique. As shown in Figures 8A–D, compared to the BLA group, the ADM group displayed increased levels of Asp and Glu within 480 min, while the SCH group’s levels of Asp and Glu approached those of the BLA group after treatment. Asp and Glu are excitatory neurotransmitters closely associated with learning and memory abilities (Francis, 2003; Teleanu et al., 2022). Elevated levels of Glu and Asp in the hippocampus of AD rats can lead to the over-activation of Glu and N-methyl-D-aspartate (NMDA) receptors (Armada-Moreira et al., 2020), the influx of Ca^2+^, and induced excitotoxicity, resulting in neuronal cell death and impaired memory and learning abilities in rats.

The hippocampus of AD rats exhibited reduced levels of Tau and Ach. Tau mitigates nervous system damage in AD rats by reducing phosphorylated tau protein levels, thereby countering excitotoxicity, oxidative stress, and apoptosis (Menzie et al., 2014). Ach, a pivotal neurotransmitter in the central nervous system, directly influences physiological processes such as learning and memory (Chen et al., 2022). Following treatment, Asp, Glu, Ach, and Tau levels in SCH-treated rats approached those of the BLA group, indicating Schisandra chinensis lignans’ potential to rectify disrupted neurotransmitter levels in the rat hippocampus.

### 3.5 Pharmacokinetic analysis

Next, all the rats were administrated with Schisandra lignans and the concentrations of each lignan component within the hippocampal microdialysate were gauged at different time points by UPLC-TQ-MS. Of the 15 lignan components, 8 were identified in the hippocampal dialysate. Supplementary Table S3 and Figures 8E–L reveal that the *C*_*max*_, *AUC*_*0-t*_, and *AUC*_*0-∞*_ of the ADM group surpassed those of the BLA group (*p* < 0.05), with the drug concentration-time curve surpassing that of both the BLA and SCH groups, attributed to a disrupted blood-brain barrier balance in AD rats, leading to increased Schisandra chinensis lignans absorption. Significant differences (*p* < 0.05) emerged in *T*_*max*_, *t*_*1/2*_, and *MRT*_*0-∞*_ between the ADM and BLA groups, reflecting variances in absorption and metabolism due to structural dissimilarities. Following treatment, the concentrations of most lignans in the SCH group exhibited a trend towards restoration, indicating that Schisandra chinensis can ameliorate brain impairment in AD rats, thereby bringing their absorption levels closer to those observed in healthy rats. Gomisin D, gomisin J, benzoylgomisin H, gomisin G, deoxyschisandrin, schisandrin B, and schisandrin C were undetected, potentially due to their low content in the purified solution or their excessive lipid solubility impeding the penetration of the aqueous probe membrane (Shannon et al., 2013).

### 3.6 Fitting of pharmacokinetic and pharmacodynamic parameters

#### 3.6.1 The fitting of PK and PD parameters

The time-average concentration values for the eight lignan concentrations were input into WinNonLin for compartmental model fitting. The Akaike information criterion (AIC) value (Katsube et al., 2008), Bayesian information criterion (BIC) value (Combes et al., 2013), and Coefficient of Variation (CV) (Castel-Branco et al., 2005) were employed as fitting criteria for the corresponding PK parameters. The Sigmoid-Emax model (van Steeg et al., 2007) was utilized to extract pharmacodynamic parameters, utilizing Asp, Glu, Tau, and Ach as indicators of pharmacodynamic response.

#### 3.6.2 Establishment of PK-PD model

**FIGURE 9:**
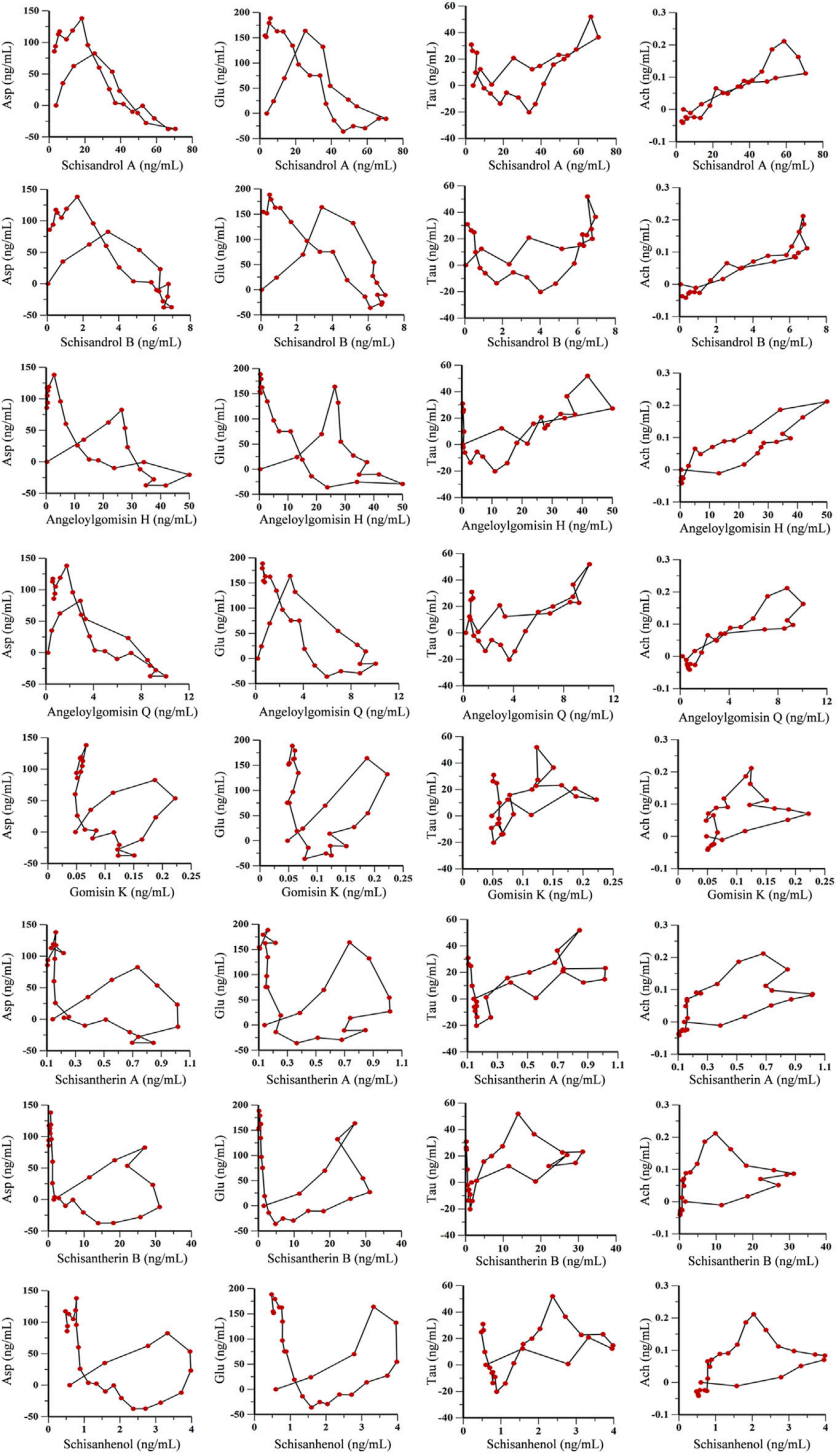
PK-PD correlation analysis of 8 lignans and 4 neurotransmitters in AD model rats.

**FIGURE 10:**
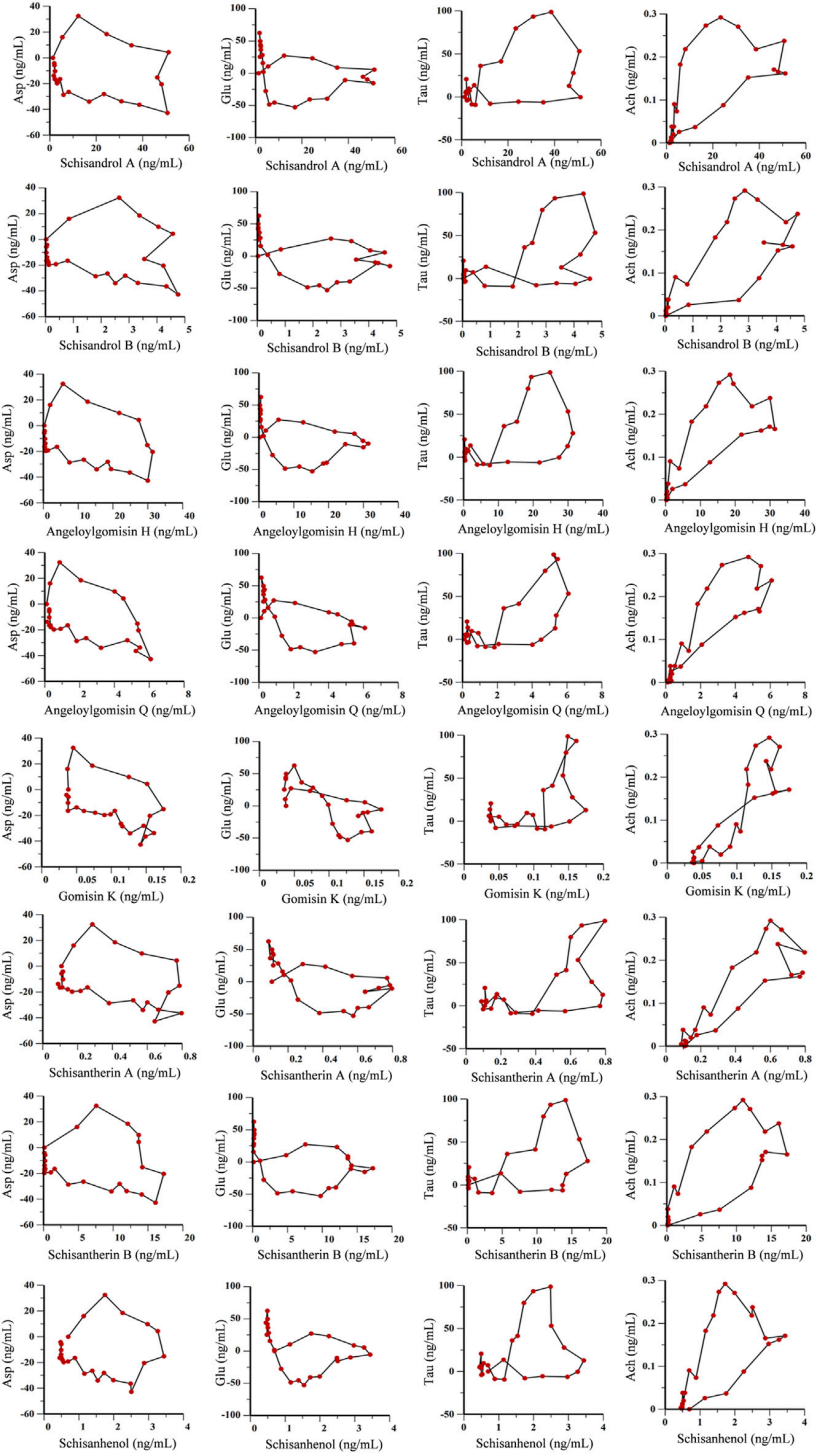
PK-PD correlation analysis of 8 lignans and 4 neurotransmitters in hippocampus of AD rats treated with purified Schisandra lignans.

Time, average concentration, and average effect values were integrated into WinNonLin software for data processing. Concentration, effect, and time curves were fitted by combining PK and PD parameters. PK-PD binding model parameters are summarized in Supplementary Table S4, and PK-PD correlation analysis diagrams for the ADM and SCH groups are depicted in Figure 9 and Figure 10.

The PK-PD binding model parameters in Supplementary Table S4 revealed that the E_0_ values for lignan components Asp and Glu were higher in the ADM group than in the SCH group, indicating elevated initial levels of Asp and Glu in AD rats compared to SCH rats. Furthermore, the ADM group’s I_max_ and IC_50_ values exceeded those of the SCH group, implying that Schisandra chinensis lignans in the ADM group have lower receptor affinity, greater intrinsic activity, and more potent effects, which might be attributed to the abnormal elevation of excitatory neurotransmitters Asp and Glu in AD rat brains, whereas Schisandra chinensis lignans ameliorated these irregularities. Compared to the ADM group, the E_0_ and E_max_ values for Tau and Ach in the SCH group were significantly elevated. Most lignans’ EC_50_ values fitted with Tau and Ach decreased, reflecting heightened receptor affinity and improved Tau and Ach production in AD rats’ post-treatment.

As shown in Figures 9, 10, the fitting curves for lignan components with Asp and Glu in both ADM and SCH groups showed a clockwise lag, and the pharmacological effects were negatively correlated with drug concentration. In contrast, tau and acetylcholine trends exhibited a counterclockwise lag with lignan concentration, signifying a positive concentration-effect correlation. However, the effect was substantially delayed compared to the concentration, suggesting that lignans do not directly affect nerve cell neurotransmitter release or inhibition but indirectly modulate neurotransmitter levels via alternative pathways to achieve therapeutic effects. Moreover, the PK-PD correlation curve demonstrates that compared to the ADM group, Asp and Glu levels in the SCH group slightly decreased at the end, indicating a significant reduction in Asp and Glu levels in AD model rats following Schisandra chinensis lignans treatment. Furthermore, the peak values for Tau and Ach effects in the SCH group exceeded those of the ADM group, indicating enhancement of Tau and Ach levels, suggesting that Schisandra chinensis lignans can regulate neurotransmitter levels in AD patient brains, potentially stimulating the release of Tau and Ach.

## 4 Discussion

In previous experiments, we have found that lignan constituents have the effect of improving AD. In this study, the UPLC method was used to further investigate 15 constituents in the purified Schisandra chinensis lignans and to determine the content of each constituent. Furthermore, this study used the Aβ25-35 protein to establish AD rat model and explored the pharmacodynamic mechanism of Schisandra lignans in treating AD. Behavioral assessments showed improved learning and memory in AD rats after treatment. Additionally, pharmacokinetic results revealed that the occurrence of AD led to increased uptake of lignans in the rat hippocampus, subsequently rectified by Schisandra lignans administration. PK indicators were selected from the identified lignan components in the hippocampus, and neurotransmitters served as PD indicators to establish a PK-PD model. Significantly, this model described the three-dimensional relationship among time, concentration, and effects of Schisandra chinensis lignans in AD treatment. Importantly, PK-PD correlation analysis indicated a complex pattern of delayed drug effects in relation to concentration. This study contributes a theoretical framework for exploring Schisandra lignans’ mechanism of action in AD treatment. Compared with our previous studies (Wei et al., 2019a), the MD-LC-TQ-MS method utilized in this study enabled us to achieve long-term continuous sampling in the hippocampus of awake rats, thus avoiding differences between individuals. At the same time, the technology can reduce the number of experimental animals and enhance the reliability of the results. Moreover, MD-LC-TQ-MS has the capability to further elucidate the therapeutic effects and pharmacokinetic characteristics of drugs *in vivo*, while accurately monitoring real-time changes in endogenous active substances.

*In vitro* researches have reported that schisandrin lignans can reduce the levels of pro-inflammatory factors such as IL-1β, IL-6 and NO in microglia induced by Aβ1-42, inhibit the activity and phosphorylation of NF-κB, IκBα, p38, JNK and ERK proteins, and activate the NF-κB/MAPK signalling pathway to reduce neuroinflammation in microglia (Gubandru et al., 2013; Yang et al., 2019). In addition, the Schisandra lignans have the ability to promote cell proliferation and inhibit apoptosis through PI3K/Akt pathway activation, and play a protective role in hypoxia-induced PC12 cell injury (Zhang et al., 2018b). Through pathological examination of AD rats after treatment with schisandrin lignans, we found that schisandrin lignans reduced excessive activation of microglia and astrocytes and restored the level of neurotransmitters in the hippocampus. This result is in line with prior research findings.

Asp and Glu are the main excitatory neurotransmitters in the brain and are closely related to learning and memory (Watkins and Evans, 1981). Studies have found that the reduction of glutamate transporter in the hippocampus and the production of Aβ protein in the brain of AD patients reduce the activity of glutamate receptors, resulting in excessive Glu that cannot be cleared in time (Scott et al., 2011). The accumulated Glu and Asp can activate Ca^2+^ channels and make Ca^2+^ flow in large quantities, thus triggering excitotoxicity and causing nerve cell death. It is manifested as lack of learning and memory ability. Tau mainly involved in the process of energy metabolism (Wen et al., 2019), and has potential improvement effects on some neurological disorders and cardiovascular diseases (Jakaria et al., 2019). Tau in AD patients can directly bind oligomeric Aβ protein, reduce the level of phosphorylated tau protein, and reduce excitotoxicity, anti-oxidation, anti-apoptosis and other mechanisms to jointly build neuroprotective function (Jahanshahi et al., 2021). Ach plays a crucial role in both the central nervous system and peripherals, and its level directly affects physiological processes such as learning, memory, sleep and awakening (Ferreira-Vieira et al., 2016).

On this basis, this study used microdialysis technology combined with PK-PD analysis to fit the three-dimensional time-concentration-drug effect relationship of Schisandra lignans in the treatment of AD, and to further explore the role of lignans in regulating neurotransmitter levels. The fitting curves for lignan components with Asp and Glu in both ADM and SCH groups showed a clockwise lag, and the pharmacological effects were negatively correlated with drug concentration. With the gradual increase of drug concentration, the levels of Asp and Glu increased slightly and then decreased until the drug concentration reached a peak. The maximum effect was observed during the drug elimination stage. Notably, the peak level of the SCH group significantly decreased compared to the ADM group, suggesting a decrease in Asp and Glu levels following Schisandra lignans treatment. Conversely, Tau and Ach trends exhibited a counterclockwise lag with lignan concentration, signifying a positive concentration-effect correlation. As the drug concentration gradually increased, the levels of Tau and Ach first decrease slightly and then increase until the concentration of the drug reaches its peak, and the maximum effect occurs before the drug reaches its peak. Following the administration of schisandra lignans, there was a significant increase in Tau and Ach levels, suggesting that schisandra has the potential to regulate neurotransmitter levels in AD rats by promoting the release of Tau and Ach.

The results revealed that the PK-PD correlation curve for the ADM and SCH groups did not demonstrate a simple linear relationship. Instead, it exhibited a complex pattern where the effect lagged behind the drug concentration. This phenomenon arises due to the drug’s initial passage through the bloodstream before reaching the target organs to generate effects, resulting in delayed drug effects relative to blood concentration (Jahanshahi et al., 2021). It shows that lignans do not directly act on nerve cells to change the release of neurotransmitters, but indirectly regulate the level of neurotransmitters through other ways and pathways to achieve the purpose of treatment. Additionally, various factors can lead to discrepancies between drug effect and concentration in hippocampal microdialysis solution. Although the characteristics of Schisandra lignans in regulating neurotransmitter levels have been explored in this paper, more specific mechanisms of action need to be investigated further.

## Data Availability

The original contributions presented in the study are included in the article/Supplementary Material, further inquiries can be directed to the corresponding author.

## Abbreviations

AD, Alzheimer’s disease; Aβ (25–35), Amyloid beta-peptide 25–35; BLA, blank control group; ADM, model group; DPZ, positive drug donepezil group; SCH, Schisandra chinensis lignans administration group; PK, pharmacokinetics; PD, pharmacodynamics; MD-LC-TQ-MS, Microdialysis coupled with ultra-performance liquid chromatography combined with triple quadruple mass spectrometry; LOD, Limit of detection; LOQ, Limit of quantitation; t_1/2_, Half time; T_max_, Peak time; C_max_, Peak concentration; AUC, Area under concentration-time the curve; MRT, Mean Retention Time; GFAP, Glial fibrillary acidic protein; Iba-1, Ionized calcium binding adapter molecule-1; NeuN, Neuronal Nuclear Protein; HE, Hematoxylin-eosin staining; NOS, Nitric oxide synthase; GSH-Px, Glutathione peroxidase; IL, Interleukin; aCSF, Artificial cerebrospinal fluid; NCA, non-compartmental model analysis; AIC, Akaike information criterion; BIC, Bayesian information criterion; CV, Coefficient of variation.
