# Supplementary material for: Establishment of a pharmacokinetics and pharmacodynamics model of Schisandra lignans against hippocampal neurotransmitters in AD rats based on microdi-alysis liquid chromatography-mass spectrometry
Source: Front Pharmacol. 2024 Mar 11;15:1342121. doi: 10.3389/fphar.2024.1342121 (PMC10961592; doi:10.3389/fphar.2024.1342121)
Supplement: Supplementary file 1 [file DataSheet1.docx]

**Establishment of a PK-PD model of Schisandra lignans against hippocampal neurotransmitters in AD rats based on microdialysis liquid chromatography-mass spectrometry**

Jinpeng Zhang^1,2†^, Xinyuan Cui^1†^, Shuo Zhao^1^, Zenghui Chang^1^, Junshuo Zhang^1^, Yufeng Chen^1^, Jiale Liu^1^, Guohao Sun^1^, Yiyuan Wang^1^, Yuanyuan Liu^1, *^

^1^ Department of Pharmaceutical Analysis, College of Pharmacy, Shandong First Medical University & Shan-dong Academy of Medical Sciences, Taian 271099, China

^2^ Qian Xi Nan Maternal and Child Care Hospital, Xingyi 562400, China

^*^ Correspondence: e-mail addresses: 15044043490@163.com

^†^ These authors contributed equally to this work.

**Table. S1 MRM parameters for 15 Schisandra lignans, IS and 4 neurotransmitters in positive mode**

| component | parent ion (m/z) | Collision Energy (V) | daughter ion (m/z) | impact energy (eV) |
| --- | --- | --- | --- | --- |
| Asp | 134.0 | 64 | 74.1^a^ | 12 |
|  |  |  | 88.0^b^ | 7 |
| Glu | 147.9 | 79 | 84.0^a^ | 16 |
|  |  |  | 129.9^b^ | 6 |
| Tau | 125.8 | 82 | 44.2^a^ | 18 |
|  |  |  | 107.9^b^ | 9 |
| Ach | 146.1 | 78 | 87.0^a^ | 12 |
|  |  |  | 60.1^b^ | 10 |
| Bifendate | 419.1 | 139 | 342.9 ^a^ | 18 |
|  |  |  | 354.9 ^b^ | 18 |
| schisandrol A | 433.1 | 194 | 415.2 ^a^ | 6 |
|  |  |  | 384.1 ^b^ | 20 |
| gomisin D | 553.1 | 284 | 507.2 ^a^ | 26 |
|  |  |  | 423.1 ^b^ | 24 |
| gomisin J | 389.1 | 139 | 287.1 ^a^ | 20 |
|  |  |  | 227.0 ^b^ | 29 |
| schisandrol B | 399.2 | 143 | 368.2 ^a^ | 18 |
|  |  |  | 330.1 ^b^ | 21 |
| angeloylgomisin H | 523.3 | 244 | 315.1 ^a^ | 36 |
|  |  |  | 409.1 ^b^ | 31 |
| benzoylgomisin H | 545.1 | 212 | 315 ^a^ | 40 |
|  |  |  | 386 ^b^ | 30 |
| angeloylgomisin Q | 553.2 | 154 | 453.1 ^a^ | 20 |
|  |  |  | 431 ^b^ | 19 |
| gomisin G | 559.1 | 259 | 437.2 ^a^ | 24 |
|  |  |  | 371.1 ^b^ | 20 |
| gomisin K | 403.0 | 134 | 302 ^a^ | 22 |
|  |  |  | 340.2 ^b^ | 20 |
| schisantherin A | 559.2 | 146 | 371.1 ^a^ | 24 |
|  |  |  | 340.2 ^b^ | 34 |
| schisantherin B | 537.2 | 159 | 371.1 ^a^ | 23 |
|  |  |  | 437 ^b^ | 19 |
| schisanhenol | 403.1 | 154 | 340.2 ^a^ | 20 |
|  |  |  | 302.1 ^b^ | 24 |
| deoxyschisandrin | 417.1 | 149 | 316.1 ^a^ | 20 |
|  |  |  | 300.9 ^b^ | 33 |
| schisandrin B | 401.1 | 134 | 300.2 ^a^ | 23 |
|  |  |  | 386.2 ^b^ | 22 |
| schisandrin C | 385.1 | 138 | 285.0 ^a^ | 20 |
|  |  |  | 227.0 ^b^ | 30 |

**Note: ^a^. quantification, ^b^.** **qualitative.**

**Table. S2 Precision, accuracy and matrix effect results for 15 lignans and 4 neurotransmitters in brain dialysate (n=8)**

| component | theoretical concentration (ng/mL) | intra-day | | | inter-day | | | matrix effect (%) | |
| --- | --- | --- | --- | --- | --- | --- | --- | --- | --- |
|  |  | Observed (ng/mL) | Precision (RSD, %) | Accuracy (RE, %) | Observed (ng/mL) | Precision (RSD, %) | Accuracy (RE, %) | Mean±SD | RSD |
| schisandrol A | 3 | 3.15±0.10 | 3.20 | 5.05 | 3.07±0.07 | 2.39 | 2.29 | 102.80±6.91 | 6.72 |
|  | 40 | 39.61±3.05 | 7.70 | -0.99 | 39.51±0.60 | 1.51 | -1.23 | 99.50±7.83 | 7.87 |
|  | 80 | 83.53±2.33 | 2.79 | 4.42 | 82.47±2.60 | 3.16 | 3.09 | 102.22±4.77 | 4.67 |
| gomisin D | 0.3 | 0.31±0.03 | 8.18 | 2.17 | 0.30±0.00 | 0.79 | 1.25 | 103.00±9.30 | 9.03 |
|  | 20 | 19.74±1.86 | 9.44 | -1.28 | 20.05±0.53 | 2.63 | 0.27 | 100.16±2.66 | 2.66 |
|  | 40 | 40.38±3.52 | 8.72 | 0.96 | 40.02±0.85 | 2.12 | 0.04 | 100.54±1.70 | 1.69 |
| gomisin J | 0.3 | 0.29±0.02 | 8.36 | -3.55 | 0.29±0.01 | 4.84 | -6.31 | 98.33±5.82 | 5.92 |
|  | 20 | 20.82±1.02 | 4.90 | 4.10 | 20.77±0.11 | 0.54 | 4.24 | 97.24±4.04 | 4.16 |
|  | 40 | 39.51±3.65 | 9.24 | 1.24 | 40.65±0.99 | 2.43 | 3.11 | 99.02±2.75 | 2.78 |
| schisandrol B | 0.3 | 0.32±0.03 | 8.11 | 5.38 | 0.31±0.01 | 3.69 | 2.29 | 99.85±2.97 | 2.98 |
|  | 20 | 20.11±0.74 | 3.68 | 0.56 | 19.60±0.57 | 2.91 | -1.42 | 100.74±7.81 | 7.75 |
|  | 40 | 38.85±4.67 | 12.03 | -2.86 | 39.09±0.95 | 2.43 | 0.33 | 102.11±3.20 | 3.13 |
| angeloylgomisin H | 3 | 3.09±0.32 | 10.26 | 2.84 | 2.99±0.12 | 4.03 | -4.87 | 101.93±6.79 | 6.67 |
|  | 30 | 30.43±2.76 | 9.06 | 1.42 | 30.02±0.52 | 1.73 | 0.62 | 98.72±5.19 | 5.26 |
|  | 60 | 58.67±6.25 | 10.66 | -2.22 | 58.78±2.25 | 3.83 | 1.81 | 98.76±2.41 | 2.44 |
| benzoylgomisin H | 3 | 2.86±0.27 | 9.27 | -4.66 | 2.96±0.09 | 3.10 | -1.21 | 92.51±7.81 | 8.44 |
|  | 30 | 29.89±2.67 | 8.94 | -0.37 | 29.87±0.74 | 2.47 | -0.43 | 95.18±7.59 | 7.97 |
|  | 60 | 57.64±2.38 | 4.13 | -3.94 | 58.04±1.17 | 2.01 | -3.27 | 96.20±8.75 | 9.09 |
| angeloylgomisin Q | 0.3 | 0.31±0.02 | 6.45 | 3.05 | 0.30±0.01 | 3.24 | -0.66 | 99.67±1.27 | 1.27 |
|  | 20 | 20.21±1.81 | 8.96 | 1.07 | 19.52±0.67 | 3.44 | -2.38 | 99.82±4.96 | 4.97 |
|  | 40 | 40.99±3.48 | 8.49 | 2.48 | 40.86±0.48 | 1.17 | 2.14 | 99.73±2.62 | 2.62 |
| gomisin G | 0.3 | 0.31±0.03 | 8.70 | 2.04 | 0.31±0.00 | 1.46 | 1.22 | 92.27±5.50 | 5.96 |
|  | 20 | 19.42±1.41 | 7.24 | -2.92 | 19.60±0.46 | 2.36 | 0.63 | 101.67±7.84 | 7.71 |
|  | 40 | 40.69±3.31 | 8.14 | 1.73 | 39.84±2.68 | 6.73 | 5.00 | 100.24±2.81 | 2.80 |
| gomisin K | 0.03 | 0.03±0.00 | 7.70 | -1.25 | 0.03±0.00 | 3.40 | 1.48 | 103.89±6.77 | 6.52 |
|  | 10 | 9.34±0.63 | 6.79 | -6.63 | 9.58±0.25 | 2.58 | -4.35 | 104.80±1.82 | 1.74 |
|  | 20 | 19.37±2.40 | 12.38 | -3.17 | 20.25±1.45 | 7.18 | 9.62 | 99.96±1.94 | 1.94 |
| schisantherin A | 3 | 3.15±0.10 | 3.20 | 5.05 | 3.07±0.07 | 2.39 | 1.38 | 98.49±7.32 | 7.44 |
|  | 40 | 39.61±3.05 | 7.70 | -0.99 | 39.51±0.60 | 1.51 | 0.14 | 97.43±6.83 | 7.01 |
|  | 80 | 83.53±2.33 | 2.79 | 4.42 | 82.47±2.60 | 3.16 | 5.48 | 99.45±2.43 | 2.45 |
| schisantherin B | 0.3 | 0.30±0.02 | 6.76 | 0.33 | 0.31±0.01 | 1.76 | 2.34 | 99.20±9.83 | 9.91 |
|  | 20 | 19.25±1.04 | 5.41 | -3.74 | 19.19±0.12 | 0.63 | -4.07 | 106.85±7.49 | 7.01 |
|  | 40 | 39.68±3.47 | 8.73 | -0.81 | 40.27±0.52 | 1.29 | 0.67 | 97.72±1.65 | 1.68 |
| schisanhenol | 0.3 | 0.31±0.02 | 5.60 | 4.41 | 0.31±0.00 | 1.23 | 3.51 | 99.62±10.15 | 10.19 |
|  | 20 | 19.99±1.42 | 7.09 | -0.04 | 20.02±0.60 | 2.97 | 0.08 | 96.38±2.74 | 2.84 |
|  | 40 | 39.31±3.85 | 9.80 | -1.74 | 39.76±0.86 | 2.17 | -0.60 | 98.74±2.17 | 2.19 |
| deoxyschisandrin | 0.3 | 0.33±0.01 | 3.97 | 9.90 | 0.33±0.00 | 1.06 | 9.44 | 102.21±9.64 | 9.43 |
|  | 20 | 19.99±1.56 | 7.81 | -0.06 | 20.14±0.41 | 2.01 | 0.68 | 104.91±7.76 | 7.39 |
|  | 40 | 39.46±4.03 | 10.20 | -1.36 | 39.61±1.46 | 3.68 | -0.98 | 100.16±2.49 | 2.48 |
| schisandrin B | 0.3 | 0.31±0.03 | 8.48 | 2.09 | 0.30±0.00 | 1.03 | 0.92 | 99.02±8.00 | 8.08 |
|  | 20 | 20.10±1.19 | 5.90 | 0.49 | 20.07±0.36 | 1.79 | 0.34 | 100.39±4.70 | 4.68 |
|  | 40 | 38.98±2.46 | 6.31 | -2.56 | 38.35±1.89 | 4.94 | -4.13 | 100.20±2.20 | 2.20 |
| schisandrin C | 3 | 3.00±0.22 | 7.48 | -0.09 | 2.99±0.05 | 1.67 | -0.20 | 95.55±12.71 | 13.30 |
|  | 30 | 30.75±2.30 | 7.47 | 2.50 | 30.53±0.32 | 1.06 | 1.76 | 104.22±6.74 | 6.47 |
|  | 60 | 59.10±5.52 | 9.33 | -1.50 | 59.59±1.87 | 3.13 | -0.68 | 98.71±2.81 | 2.85 |
| Asp | 30 | 29.77±2.08 | 6.97 | -0.78 | 29.80±0.92 | 3.09 | -0.65 | 93.76±1.27 | 1.35 |
|  | 120 | 123.29±6.40 | 5.19 | 2.74 | 122.86±2.33 | 1.90 | 2.38 | 99.88±1.74 | 1.74 |
|  | 240 | 231.90±6.42 | 2.77 | -3.37 | 235.02±1.52 | 0.65 | -2.07 | 98.80±0.98 | 1.00 |
| Glu | 60 | 59.19±3.17 | 5.36 | -1.36 | 59.11±0.61 | 1.03 | -1.49 | 99.97±5.65 | 5.65 |
|  | 160 | 163.65±9.70 | 5.93 | 2.28 | 165.60±1.70 | 1.03 | 3.50 | 97.58±1.89 | 1.93 |
|  | 320 | 339.78±11.72 | 3.45 | 6.18 | 334.53±4.72 | 1.41 | 4.54 | 100.51±2.25 | 2.24 |
| Tau | 60 | 58.34±4.25 | 7.29 | 2.77 | 57.44±0.37 | 0.64 | -4.27 | 98.30±2.06 | 2.10 |
|  | 160 | 163.13±13.10 | 8.03 | 1.96 | 164.50±1.19 | 0.72 | 2.81 | 99.33±3.30 | 3.32 |
|  | 320 | 322.18±11.38 | 3.53 | 0.68 | 320.44±2.13 | 0.67 | 0.14 | 99.24±1.36 | 1.37 |
| Ach | 0.15 | 0.16±0.00 | 0.81 | 6.92 | 0.16±0.00 | 0.40 | 7.29 | 101.17±7.72 | 7.63 |
|  | 8 | 8.24±0.59 | 7.12 | 2.94 | 8.13±0.11 | 1.35 | 1.66 | 97.65±2.16 | 2.21 |
|  | 16 | 17.31±0.38 | 2.19 | 8.18 | 17.12±0.05 | 0.31 | 7.01 | 97.11±2.48 | 2.56 |

**Table. S3 Pharmacokinetic parameters of 8 lignans in hippocampus of rats in different groups (n=8, mean±SD)**

| Analytes | Group | t_1/2_ (min) | T_max (_min) | C_max (_ng/mL) | AUC_0-t_ (min·ng/mL) | AUC_0-∞_ (min·ng/mL) | MRT_0-∞_ (min) |
| --- | --- | --- | --- | --- | --- | --- | --- |
| schisandrol A | SHA | 83.67±29.23 | 160.00±14.14 | 43.61±3.17 | 6954.84±905.76 | 7326.16±962.82 | 208.48±12.52 |
|  | ADM | 46.49±14.62* | 168.00±10.95 | 71.83±10.44** | 14795.06±1328.20** | 15009.33±1374.15** | 207.95±7.31 |
| schisandrol B | SHA | 43.20±2.19 | 164.00±38.47 | 4.06±0.36 | 646.25±43.55 | 649.22±43.65 | 156.00±5.33 |
|  | ADM | 45.61±14.78 | 168.00±10.95 | 71.83±10.44** | 14446.14±858.71** | 14656.46±1003.64** | 210.19±7.21** |
| angeloylgomisin H | SHA | 39.17±15.46 | 148.00±10.95 | 26.03±4.16 | 3567.81±498.68 | 3595.46±493.06 | 173.34±4.81 |
|  | ADM | 32.76±5.08 | 152.00±50.20 | 48.84±13.03* | 8329.57±567.35** | 8348.68±586.20** | 166.04±5.48 |
| angeloylgomisin Q | SHA | 43.63±26.61 | 152.00±10.95 | 3.95±0.60 | 674.88±81.10 | 688.80±80.46 | 182.91±13.66 |
|  | ADM | 65.62±8.30 | 164.00±32.86 | 11.06±0.99** | 1942.39±109.23** | 2005.82±105.09** | 208.81±7.93** |
| gomisin K | SHA | 204.57±27.24 | 176.00±16.73 | 0.10±0.01 | 25.68±1.61 | 36.01±2.13 | 380.60±29.01 |
|  | ADM | 233.75±109.15 | 96.00±35.78** | 0.23±0.03** | 47.31±3.51** | 64.96±11.41** | 365.00±96.57 |
| schisantherin A | SHA | 111.12±8.93 | 168.00±17.89 | 0.58±0.03 | 123.56±4.56 | 139.78±4.97 | 257.61±15.57 |
|  | ADM | 96.52±25.64 | 108.00±10.95** | 1.14±0.06** | 208.04±10.27** | 222.49±11.37** | 198.08±11.19** |
| schisantherin B | SHA | 52.76±7.79 | 136.00±8.94 | 14.95±1.41 | 2155.12±227.63 | 2172.13±236.34 | 165.92±8.33 |
|  | ADM | 41.19±12.09 | 100.00±34.64 | 32.59±5.13** | 4575.79±477.56** | 4586.33±477.04** | 118.95±8.79** |
| schisanhenol | SHA | 148.87±14.32 | 156.00±16.73 | 2.96±0.23 | 610.63±31.21 | 713.81±24.99 | 258.63±15.49 |
|  | ADM | 123.05±11.63* | 96.00±16.73** | 4.38±0.33** | 847.04±66.66** | 939.38±63.98** | 216.58±5.69** |

**Note: Compared with SHA group，*p<0.05，**p<0.01。**

**Table. S4 PK-PD parameters of 8 lignans and 4 amino acids in hippocampus dialysate**

| PK-PD  parameters | Asp | | Glu | | Tau | | Ach | |
| --- | --- | --- | --- | --- | --- | --- | --- | --- |
|  | ADM | SCH | ADM | SCH | ADM | SCH | ADM | SCH |
| schisandrol A |  |  |  |  |  |  |  |  |
| IC_50_/EC_50_（ng/mL） | 37.2351 | 14.0074 | 37.558 | 20.4304 | 39.9417 | 24.8908 | 29.9286 | 19.0353 |
| E_0_（ng/mL） | 86.2893 | 16.9404 | 126.387 | 28.7795 | 23.1654 | 62.5123 | 0.113751 | 0.198335 |
| I_max_/E_max_（ng/mL） | 112.7 | 55.0163 | 152.375 | 65.5608 | 20.6599 | 60.2622 | 0.13846 | 0.179276 |
| schisandrol B |  |  |  |  |  |  |  |  |
| IC_50_/EC_50_（ng/mL） | 4.39639 | 1.5236 | 4.37711 | 2.20957 | 4.81951 | 2.69243 | 3.55672 | 2.05661 |
| E_0_（ng/mL） | 86.3236 | 16.9398 | 125.68 | 28.782 | 23.1666 | 62.515 | 0.113809 | 0.198318 |
| I_max_/E_max_（ng/mL） | 112.716 | 54.8866 | 214.098 | 65.5857 | 20.673 | 60.2656 | 0.138604 | 0.179257 |
| angeloylgomisin H |  |  |  |  |  |  |  |  |
| IC_50_/EC_50_（ng/mL） | 21.9568 | 9.18199 | 22.1631 | 13.3225 | 23.9549 | 16.1729 | 16.5459 | 12.275 |
| E_0_（ng/mL） | 86.5095 | 16.9376 | 126.405 | 28.7982 | 23.1616 | 62.5147 | 0.114514 | 0.198188 |
| I_max_/E_max_（ng/mL） | 113.032 | 54.4999 | 154.429 | 65.7441 | 20.6654 | 60.2652 | 0.139889 | 0.179102 |
| angeloylgomisin Q |  |  |  |  |  |  |  |  |
| IC_50_/EC_50_（ng/mL） | 5.00931 | 1.95917 | 5.0597 | 2.80108 | 5.36738 | 3.37124 | 3.95936 | 2.56289 |
| E_0_（ng/mL） | 86.3359 | 16.9363 | 126.392 | 28.7859 | 23.1761 | 62.5156 | 0.113818 | 0.197933 |
| I_max_/E_max_（ng/mL） | 112.728 | 54.0294 | 152.924 | 65.7749 | 20.6881 | 60.266 | 0.138622 | 0.178758 |
| gomisin K |  |  |  |  |  |  |  |  |
| IC_50_/EC_50_（ng/mL） | 0.105001 | 0.0823722 | 0.104373 | 0.1104 | 0.114013 | 0.128529 | 0.0844244 | 0.103216 |
| E_0_（ng/mL） | 86.0289 | 16.9519 | 126.357 | 28.5893 | 23.1579 | 62.5222 | 0.112934 | 0.198005 |
| I_max_/E_max_（ng/mL） | 111.901 | 54.8035 | 150.163 | 64.609 | 20.6608 | 60.2705 | 0.137593 | 0.178709 |
| schisantherin A |  |  |  |  |  |  |  |  |
| IC_50_/EC_50_（ng/mL） | 0.444088 | 0.310322 | 0.485777 | 0.439889 | 0.534221 | 0.527031 | 0.358387 | 0.402679 |
| E_0_（ng/mL） | 86.4045 | 16.9368 | 126.406 | 28.7686 | 23.1617 | 62.5166 | 0.114889 | 0.197851 |
| I_max_/E_max_（ng/mL） | 112.742 | 53.9706 | 154.566 | 65.6944 | 20.6655 | 60.2666 | 0.140638 | 0.178629 |
| schisantherin B |  |  |  |  |  |  |  |  |
| IC_50_/EC_50_（ng/mL） | 11.0195 | 5.18879 | 10.7004 | 7.53579 | 12.8672 | 9.19058 | 8.39429 | 7.04318 |
| E_0_（ng/mL） | 86.5066 | 16.9407 | 126.379 | 28.7738 | 23.1653 | 62.515 | 0.114824 | 0.198358 |
| I_max_/E_max_（ng/mL） | 112.943 | 55.0686 | 152.236 | 65.5146 | 20.6706 | 60.2656 | 0.140671 | 0.179302 |
| schisanhenol |  |  |  |  |  |  |  |  |
| IC_50_/EC_50_（ng/mL） | 1.91311 | 1.09997 | 1.90289 | 1.58902 | 2.12765 | 1.93324 | 1.442 | 1.47092 |
| E_0_（ng/mL） | 86.5075 | 16.9386 | 126.397 | 28.7925 | 23.1581 | 62.5149 | 0.114646 | 0.19825 |
| I_max_/E_max_（ng/mL） | 113.007 | 54.6221 | 153.553 | 65.6817 | 20.66 | 60.2654 | 0.140223 | 0.179178 |
